# Mucosal and systemic SIV-specific cytotoxic CD4^+^ T cell hierarchy in protection following intranasal/intramuscular recombinant pox-viral vaccination of pigtail macaques

**DOI:** 10.1038/s41598-019-41506-5

**Published:** 2019-04-05

**Authors:** Mayank Khanna, Ronald J. Jackson, Sheilajen Alcantara, Thakshila H. Amarasena, Zheyi Li, Anthony D. Kelleher, Stephen J. Kent, Charani Ranasinghe

**Affiliations:** 1 Molecular Mucosal Vaccine Immunology Group, Department of Immunology and Infectious Disease, The John Curtin School of Medical Research, The Australian National University, Canberra ACT, 2601 Australia; 2 Department of Microbiology and Immunology, Peter Doherty Institute, University of Melbourne, Melbourne, VIC 3010 Australia; 3 Immunovirology and Pathogenesis Program, Kirby Institute, University of New South Wales, Sydney, NSW 2052 Australia; 4 Present Address: Department of Microbiology, Immunology and Parasitology, Louisiana State University Health Sciences Center, New Orleans, LA 70112 USA

## Abstract

A HIV vaccine that provides mucosal immunity is urgently needed. We evaluated an intranasal recombinant Fowlpox virus (rFPV) priming vaccine followed by intramuscular Modified Vaccinia Ankara (rMVA) booster vaccine, both expressing SIV antigens. The vaccination generated mucosal and systemic SIV-specific CD4^+^ T cell mediated immunity and was associated with partial protection against high-dose intrarectal SIV_mac251_ challenge in outbred pigtail macaques. Three of 12 vaccinees were completely protected and these animals elicited sustained Gag-specific poly-functional, cytotoxic mucosal CD4^+^ T cells, complemented by systemic poly-functional CD4^+^ and CD8^+^ T cell immunity. Humoral immune responses, albeit absent in completely protected macaques, were associated with partial control of viremia in animals with relatively weaker mucosal/systemic T cell responses. Co-expression of an IL-4R antagonist by the rFPV vaccine further enhanced the breadth and cytotoxicity/poly-functionality of mucosal vaccine-specific CD4^+^ T cells. Moreover, a single FPV-*gag/pol/env* prime was able to induce rapid anamnestic gp140 antibody response upon SIV encounter. Collectively, our data indicated that nasal vaccination was effective at inducing robust cervico-vaginal and rectal immunity, although cytotoxic CD4^+^ T cell mediated mucosal and systemic immunity correlated strongly with ‘complete protection’, the different degrees of protection observed was multi-factorial.

## Introduction

Despite the availability of highly active antiretroviral therapy (ART), human immunodeficiency virus-1 (HIV-1) remains a significant global health burden with an estimated 36.7 million people infected to date and 1.8 million new infections in 2016^1^. Lifelong ART, although effective, is associated with high costs and emergence of drug-resistant viruses, making ART less than ideal as a long-term solution^2^. A cost effective prophylactic HIV vaccine inducing both cytotoxic cellular immunity and humoral immunity for protection, is widely viewed as an essential component to a long-term solution. Since HIV preferentially targets mucosal CD4^+^ T cells, an ideal vaccine would induce effective mucosal immunity and provide immediate control of viral replication^3–10^.

Over the last two decades several heterologous prime-boost vaccine strategies, although have shown promising immune outcomes in animals, have yielded disappointing immune outcomes in human Phase I/II trials. Among these examples are our own Phase I recombinant DNA (rDNA)/recombinant Avipoxvirus fowlpox (rFPV) vaccine trial^11,12^, the HVTN 505 phase IIb trial which utilised a rDNA prime followed by a recombinant adenovirus 5 (rAd5) booster strategy^13^, and also the EV02 Phase I trial where a rDNA vaccine was followed by New York Vaccinia strain (NYVAC)^14^. Interestingly, the RV144 trial, which used four recombinant canarypox virus primes followed by two AIDSVAX® B/E boosts, is the only strategy to date that has yielded some efficacy in humans. The 31.2% protective efficacy observed was mainly associated with Fc-functional antibody responses against gp120, and also envelope-specific CD4^+^ T cell-mediated immunity^15–17^. The phase IIb STEP trial, a single rAd5 virus vector-based vaccine expressing HIV Gag-Pol and Nef antigens^18,19^, not only failed to confer protection against HIV, but exacerbated infection in men with pre-existing Ad5 immunity^20^. However, mucosal and systemic delivery of recombinant Modified Vaccinia Ankara (rMVA) and NYVAC in prime-boost modalities (i.e. rMVA/Adenovirus) have also shown to induce effective mucosal and systemic immunity in murine and non-human primates^21–25^.

The effectiveness of a HIV vaccine will likely not only depend upon the vaccine antigens but also the route of administration, cytokine milieu, timing and the vaccine vector combination^26–31^. Although HIV is a disease of the mucosae, with the gut being the primary site of CD4^+^ T cell depletion^32,33^, no mucosal viral-vector-based HIV prime-boost vaccine strategy has been clinically tested to our knowledge. Historical evidence clearly demonstrates that mucosal vaccination is the best solution for mucosal pathogens^34,35^. Designing an HIV vaccine strategy that can induce effective mucosal immunity is a high priority^27,33,36,37^. Studies in our laboratory have shown that intranasal (i.n.) rFPV prime, (a viral vector similar to canarypox virus) followed by an intramuscular (i.m.) booster with recombinant vaccinia virus (rVV) or rMVA expressing HIV antigens, induced sustained mucosal and systemic HIV-specific CD8^+^ T cell immunity^27,38^. rFPV was a useful intranasal priming delivery vector^27,37,39^ and does not cross the olfactory receptor neuron pathway^40^, similar to what has been reported with rMVA^23^. Our studies also led to the discovery that IL-13 plays a crucial role in modulating T cell avidity in a route dependent manner, where mucosal vaccination induced high avidity T cells with improved efficacy by lowering innate lymphoid cells type 2-driven IL-13 expression at the vaccination site^41^ and T cell driven IL-13 at the adaptive immune level^28,42,43^. Furthermore, an IL-4R antagonist adjuvanted (IL-4R antagonist) vaccine that transiently inhibited IL-4/IL-13 signalling via STAT6 pathway at the vaccination site^41^, was shown to induce immune responses similar to that observed in HIV elite controllers^44–46^. Specifically, resulting in enhanced mucosal and systemic high avidity/poly-functional HIV-specific CD8^+^ T cells and robust long-lived HIV Gag-specific B-cell immunity^47^. Moreover, this strategy following a gp140 Env protein booster in mice has also been shown to induce effective Env-specific antibodies (Ranasinghe *et al*. unpublished observations). Given these promising features, in the current study we evaluated the protective efficacy of IL-4R antagonist adjuvanted vaccine compared to unadjuvanted and empty vector controls, in an i.n. rFPV/i.m rMVA prime-booster modality followed by a high dose intra rectal SIV_mac251_ challenge in pigtailed macaques.

## Results

### i.n./i.m. IL-4R antagonist and unadjuvanted vaccine regimens were well tolerated and induced greater poly-functional systemic Gag-specific CD4^+^ T cell responses than CD8^+^ T cell responses

**Figure 1:**
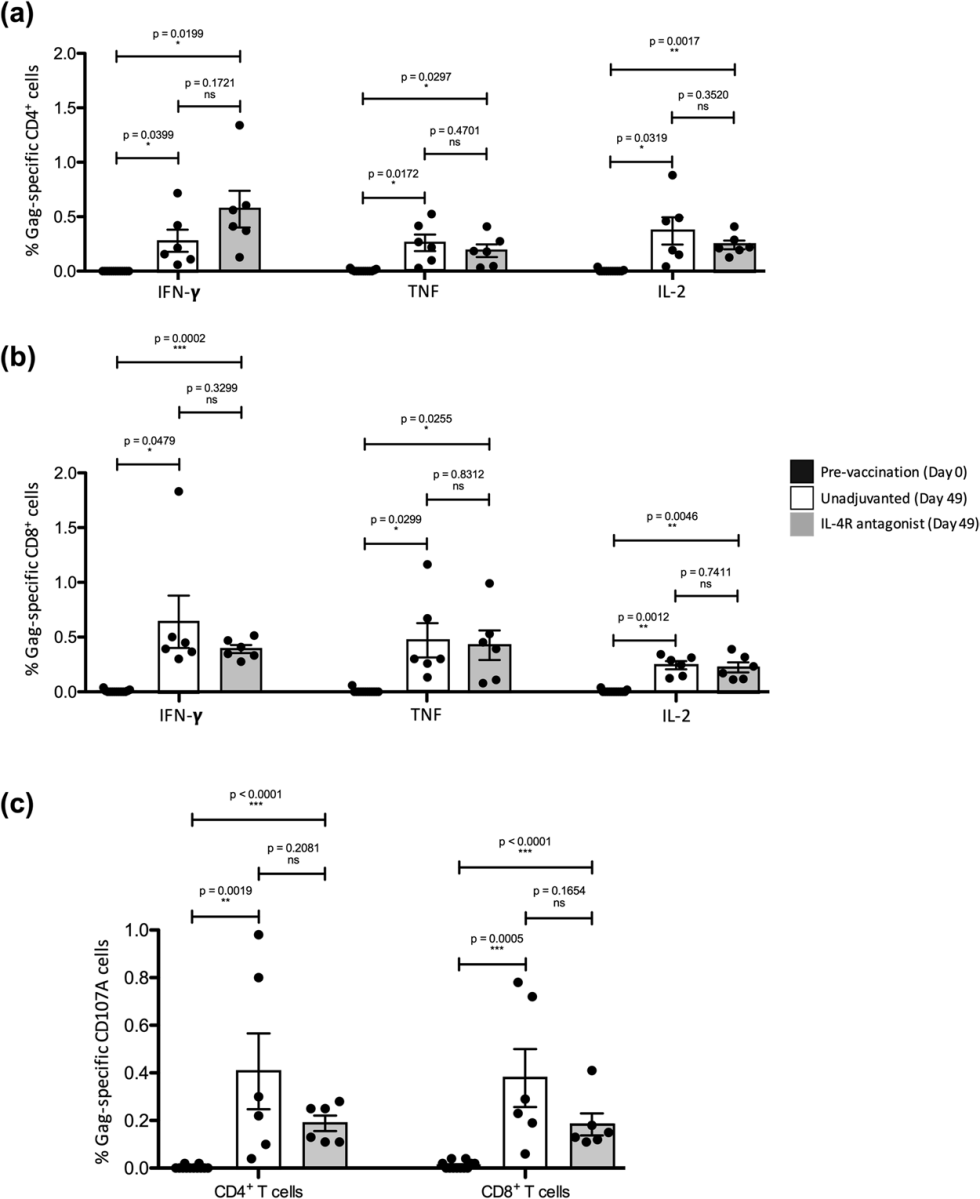
Evaluation of Gag-specific systemic CD4^+^ and CD8^+^ T cells post first rMVA booster. Freshly isolated lymphocytes from the whole blood of all fourteen animals from bleeds at day 0 (pre-vaccination) and at day 49 (after first rMVA booster), were stimulated with a 15 mer overlapping SIV Gag-specific peptide pool and DMSO and their cytokine expression was measured by multi-colour intracellular cytokine staining. Bar charts show proportion of IFN-γ, TNF and IL-2 expression by (**a**) CD4^+^ T cells and (**b**) CD8^+^ T cells from the two vaccine groups at day 49. (**c**) Cytotoxicity of systemic CD4^+^ and CD8^+^ T cells at day 49 was also determined by the quantification of CD107A expression, compared to baseline (day 0). Statistical significance was assessed using a nonparametric Student’s t test (ns – not significant), with a paired t test performed to compare respective cytokine expression at day 0 and day 49 for both vaccine groups and an unpaired t test to compare cytokine expression between the two vaccine groups.

The macaques were vaccinated as per the schedule indicated in the timeline (see Supplementary Fig. S1) with constructs listed in Table 1. The vaccines were well tolerated with no local reactions or differences in mean weight, haematology parameters, or other clinical observations across the groups (see Supplementary Fig. S2). At day 49 (7 days post first rMVA booster), elevated SIV Gag-specific IFN-γ, TNF and IL-2 expression by peripheral blood CD4^+^ and CD8^+^ T cells were observed in both unadjuvanted and IL-4R antagonist vaccine groups compared to the baseline expression (Fig. 1a,b) (also see Supplementary Fig. S3). These T cells also expressed degranulation marker CD107A, indicative of their cytotoxic potential (Fig. 1c). However, no significant differences in any of the markers were detected between the IL-4R antagonist adjuvanted and unadjuvanted vaccine groups (Fig. 1a,b) (also see Supplementary Fig. S3), unlike our murine studies^48^. IL-17, predominantly produced by Th17 cells, has previously been shown to play a key role in maintaining mucosal immunity against specific pathogens, with loss of IL-17 being linked to viral persistence^49,50^. Our results show that on an average 0.2–0.3% of CD4^+^ and CD8^+^ T cells from both the unadjuvanted and the IL-4R antagonist vaccine groups were found to express IL-17A following Gag stimulation (data not shown).

**Table 1:** Vaccine groups.

Vaccine	No. of Animals	Animal ID					
Unadjuvanted (FPV-SIV^†^/MVA-SIV^)	6	A730 ♀	9B7A ♂	55A3 ♂	A50D ♀	D2CD ♂	94D5 ♀
IL-4R Antagonist (FPV-SIV^†^ IL-4R Antagonist/MVA-SIV^ IL-4R Antagonist)	6	DD1B ♀	ECEB ♀	AAB5 ♀	9CBC ♀	DA45 ♂	F068 ♂
Empty (FPV-empty/MVA-empty)	2	B68D ♀	CE71 ♂				

^†^FPV-SIV expresses SIV *gag*, *pol* and *env*; ^MVA-SIV expresses SIV *gag* and *pol*.

**Figure 2:**
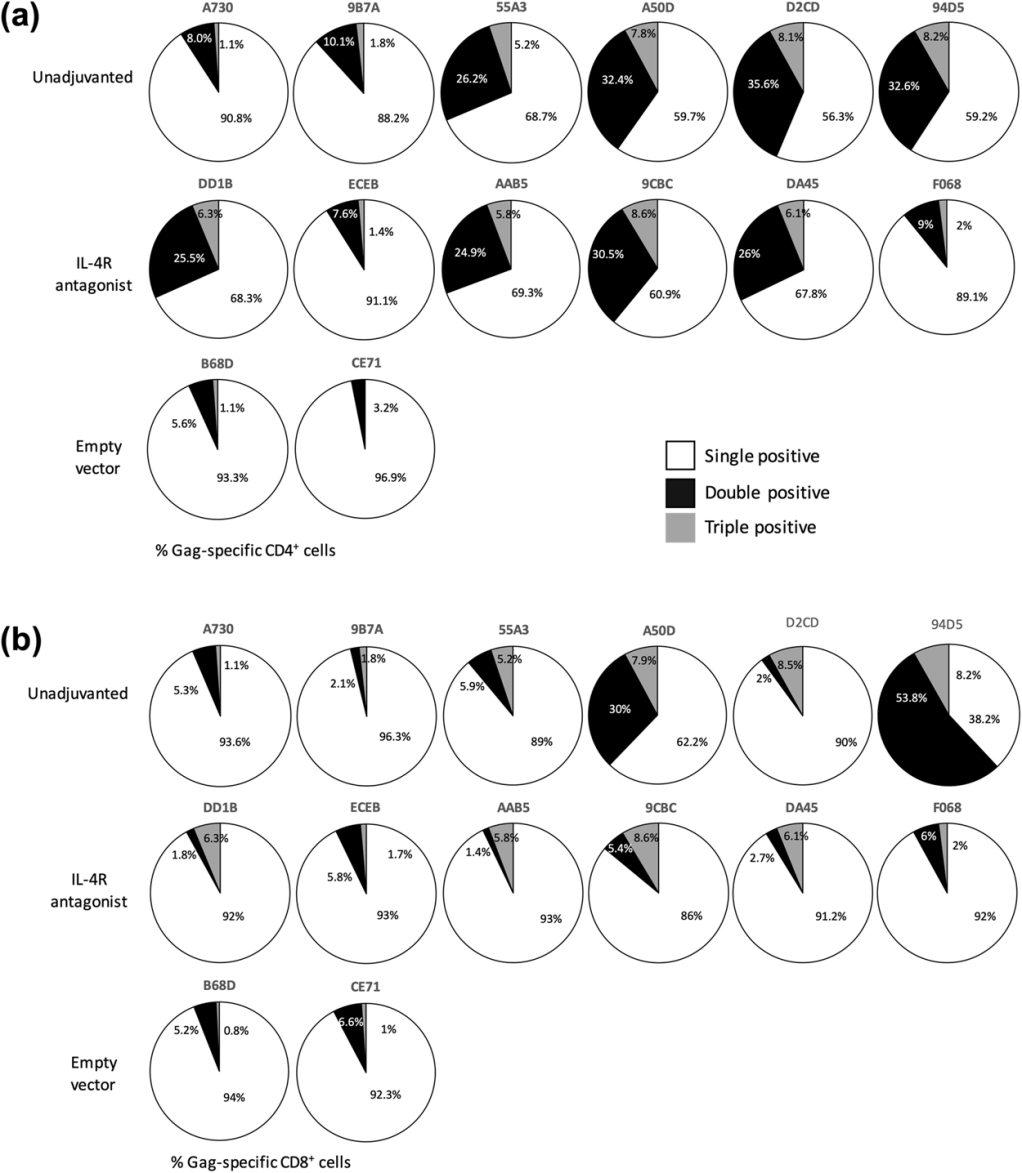
Evaluation of SIV-specific poly-functional T cells. Pie charts show the proportion of cytokine expressing Gag-specific systemic (**a**) CD4^+^ T cell and (**b**) CD8^+^ T cell populations expressing only one of the three cytokines of interest at day 49, namely IFN-γ, TNF or IL-2 – single positive (white), a combination of two cytokines – double positive (black) and all three cytokines – triple positive (grey).

Next, the quality of T cells was also assessed by determining cytokine poly-functionality, which is a hallmark of protective efficacy as seen in previous studies^48^. Eight out of 12 macaques that received the SIV vaccination showed 25% or greater proportion of SIV Gag-specific CD4^+^ T cells, producing more than one cytokine (IFNγ, TNF or IL-2) (Fig. 2a). Whereas, only 2 out of 12 macaques showed such profile at the CD8^+^ T cell level (Fig. 2b). The empty vector group displayed no or minimal poly-functionality.

### IL-4R antagonist vaccine strategy increased breadth of T cell immunity by inducing Pol-specific IL-2 expression by both CD4^+^ and CD8^+^ T cells

**Figure 3:**
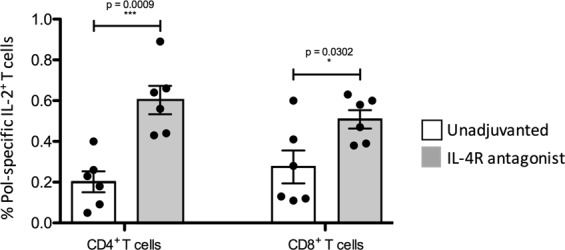
Evaluation of Pol-specific systemic CD4^+^ and CD8^+^ T cells. Freshly isolated lymphocytes from the whole blood of animals in both unadjuvanted and IL-4R antagonist vaccine groups were also stimulated with a 15 mer Pol-specific overlapping peptide pool and their cytokine expression measured by multi-colour intracellular cytokine staining. The IL-2 expression by CD4^+^ and CD8^+^ T cells from unadjuvanted and IL-4R antagonist adjuuvanted vaccines was also compared using an unpaired student’s t test.

The Pol-specific 15-mer overlapping peptide pool was used in intracellular cytokine staining assays to determine whether the unadjuvanted or the IL-4R antagonist vaccine regimes could modulate the breadth (number of important targets induced) of SIV-specific immunity. Interestingly, the macaques that received the IL-4R antagonist vaccines displayed significantly elevated systemic Pol-specific CD4^+^ and CD8^+^ T cells expressing IL-2 (Fig. 3), although the Pol-specific IFN-γ and/or TNF expression were similar between the two groups (see Supplementary Fig. S4). Furthermore, in both vaccinated groups, only 20% Pol-specific T cells were poly-functional.

### Markedly enhanced SIV Gag KP9 tetramer-specific CD8^+^ T cell responses were detected in some macaques post challenge

**Figure 4:**
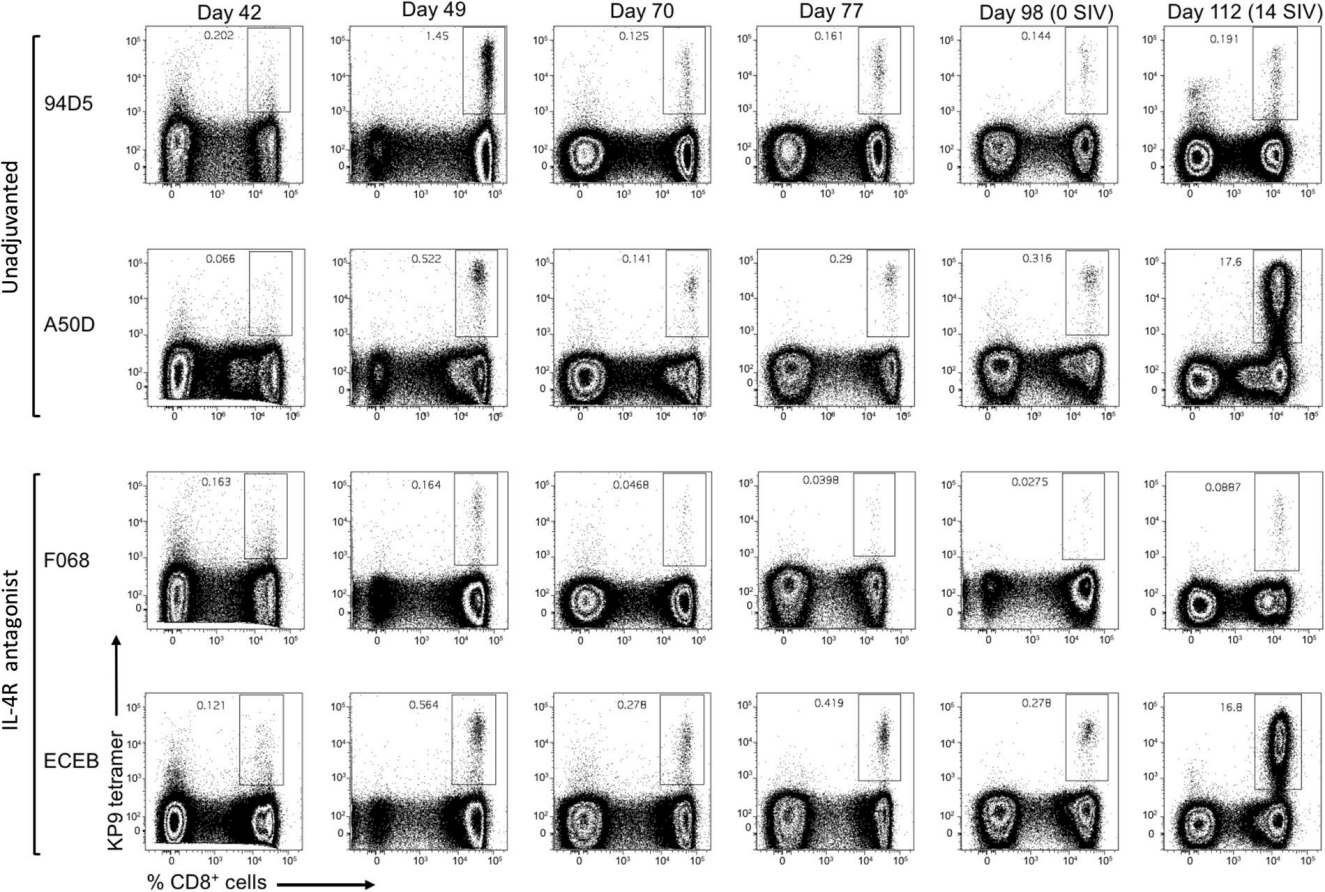
Evaluation of Gag-KP9 tetramer specific CD8 T cells pre-and post-SIV_mac251_ challenge. The flow cytometry plots indicate SIV Gag-KP9 Mane-A*084 MHC-I tetramer-specific CD8^+^ T cells assessed at various time points post immunisation (day 42, 49, 70, 77, 98) and following challenge (day 112–14 days post SIV), in peripheral blood.

The MHC I allele Mane-A*084 is expressed by a subset of outbred pigtail macaques. In this study two macaques that were Mane-A*084 positive were included in each vaccine group and SIV Gag KP9 specific responses were assessed using a Mane-A*084/KP9 tetramer^51–53^ in whole blood at different time points and post SIV_mac251_ challenge. Readily detectable KP9-specific CD8^+^ T cell responses were observed in all 4 vaccinated macaques 7 days post first rMVA booster vaccination (Day 49) (Fig. 4). The second rMVA booster (Day 77) resulted in only a modest increase in the magnitude of the KP9 specific CD8^+^ T cell immunity, suggesting that the second booster was minimally beneficial. Fourteen days post high-dose intrarectal SIV_mac251_ challenge (day 112), two of the 4 macaques (from each vaccine group) showed markedly enhanced KP9-specific CD8^+^ T cells at levels of up to ~17% of all CD8^+^ T cells (Fig. 4). The other 2 macaques showed minimally enhanced KP9-specific CD8^+^ T cells after SIV_mac251_ challenge. Interestingly, these two macaques showed no detectable viremia post challenge (see below). Note that our previous studies with 44 pig-tailed macaques have shown that the presence of Manu-A084 was not associated with a significantly improved control of SIV^54^, a finding that was also shown by another study using 32 pig-tailed macaques^55^.

### Following high dose intrarectal SIV_mac251_ challenge, three vaccinated macaques showed no viremia

**Figure 5:**
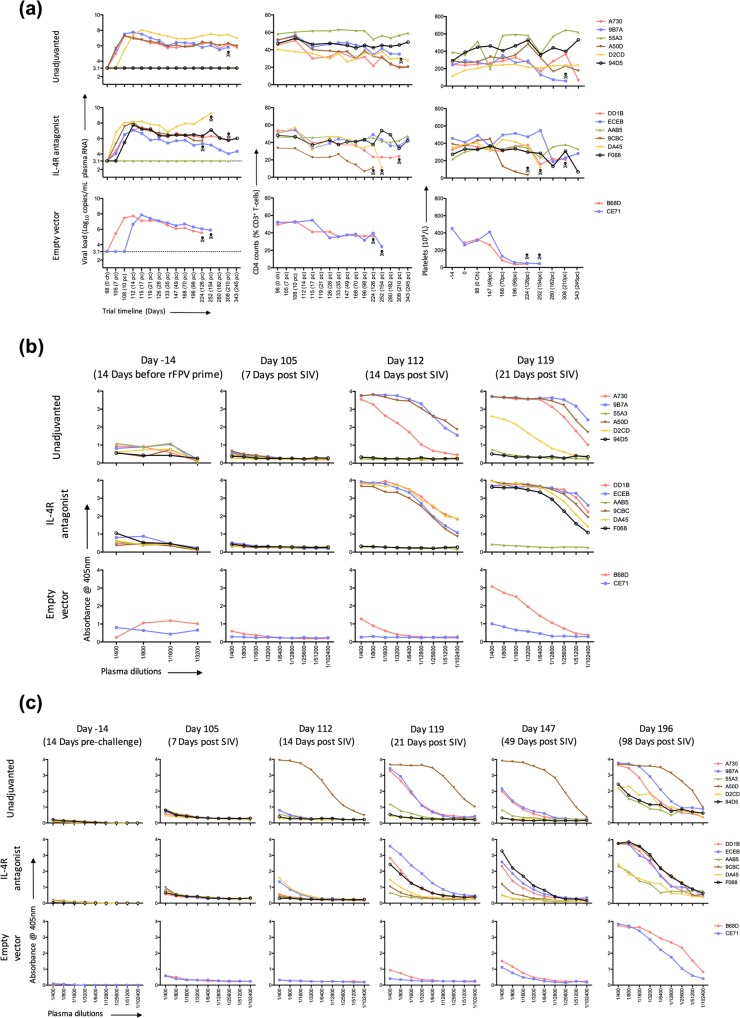
Evaluation of viral load, CD4^+^ T cell counts, platelet counts and of Gag- and Env-specific humoral responses. All fourteen macaques were bled at regular intervals as per timeline pre and post high dose intrarectal SIV_mac251_ challenge and (**a**) systemic viral load (left panel) measured by determining the Log_10_ copies/mL plasma SIV_mac239_ RNA, proportion of circulating CD4^+^ T cells (middle panel) and platelet counts per litre of blood (right panel) measured. The macaques are grouped in their respective vaccine groups, with “” representing the time point when an animal was euthanized. (**b**) Env- and (**c**) Gag-specific total IgG in the plasma of all fourteen macaques pre-and post SIV_mac251_ challenge were determined using ELISA as per described in materials and Methods. Graphs show absorbance readings at 405 nm for plasma dilutions from 1/400–1/102,400.

The protective efficacy was evaluated by determining the viral load (Log_10_ copies/mL plasma SIV_mac251_ RNA) after a high dose intrarectal SIV_mac251_ challenge (Fig. 5a) as indicated in methods. Three macaques, two in the unadjuvanted group (55A3, 94D5) and one in the IL-4R antagonist group (AAB5) showed no detectable plasma SIV_mac251_ RNA over the course of the trial. These 3 animals also retained total peripheral CD4^+^ T cells and platelet counts (parameters that typically decline during progressive SIV infection^56^) (Fig. 5a). Animals in the empty vector group were euthanized as per ethics guidelines at 126/154 days post challenge due to high SIV RNA levels and declining CD4^+^ T cell and platelet counts (Fig. 5a). In the 9 viremic macaques across the unadjuvanted and antagonist vaccine groups, fluctuating levels of plasma SIV_mac251_ RNA levels, CD4^+^ T cell and platelet counts were observed and 4 were euthanised during the follow up period (Fig. 5a).

### Single FPV-SIV *gag/pol/env* prime induced an anamnestic Env-specific antibody responses following SIV_mac251_ challenge

As modest protection in the RV144 trial was associated with Env-specific antibodies^15^, in this study we also evaluated SIV_mac239/251_ Env- and Gag-specific responses (Fig. 5b,c). Although Env-specific IgG antibody responses were not clearly detectable immediately post FPV-SIV *gag/pol/env* vaccination, an anamnestic response was detected by 14 days post SIV_mac251_ challenge in the vaccinated groups, unlike the empty vector control (Fig. 5b). This was unexpected as Env antigen was only a component of the intranasally administered rFPV prime, and not the rMVA boosters.

At 21 days post challenge elevated SIV_mac239/251_ Env-specific IgG responses were detected in all of the viremic vaccinees, including some modest responses in the two macaques that received the empty vector. However, at 21 or 49 days post SIV challenge, the three macaques with no detectable SIV viremia (Fig. 5a) also lacked any SIV Env-specific antibodies.

### i.n. rFPV/i.m rMVA prime-boost vaccination strategy primed for Gag-specific antibodies following SIV_251_ challenge

In the context of SIV_mac239/251_ Gag-specific IgG antibody responses, macaque A50D in the unadjuvanted vaccine group showed the highest antibody level compared to all the other macaques at 14 and 21 days post challenge (Fig. 5c). Although, by 21 days post challenge, macaques A730 and 9B7A in the unadjuvanted group showed progressive SIV_mac239/251_ Gag-specific IgG responses, low-to-no responses were observed in macaques 94D5, D2CD and 55A3 (Fig. 5c). Animals in the antagonist vaccine group showed varying levels of SIV_mac239/251_ Gag-specific IgG responses, with AAB5 and 9CBC being the lowest. As expected, no SIV_mac239/251_ Gag-specific IgG responses were detected in the macaques that received the empty vector vaccines at day 21 post challenge. Similar to SIV_mac239/251_ Env-specific responses, by day 49, all but 94D5, 55A3 and AAB5 expressed SIV_mac239/251_ Gag-specific IgG antibodies (Fig. 5c). Overall, at 21 days post challenge time interval, the SIV_mac239/251_ Gag-specific endpoint titres were much lower compared to SIV_mac239/251_ Env, with A50D being the only macaque showing consistently high Gag and Env antibody titres (51,200 and 102,400 respectively) (see Supplementary Fig. S5).

### Poly-functional Gag-specific systemic CD4^+^ T cells were associated with protection

**Figure 6:**
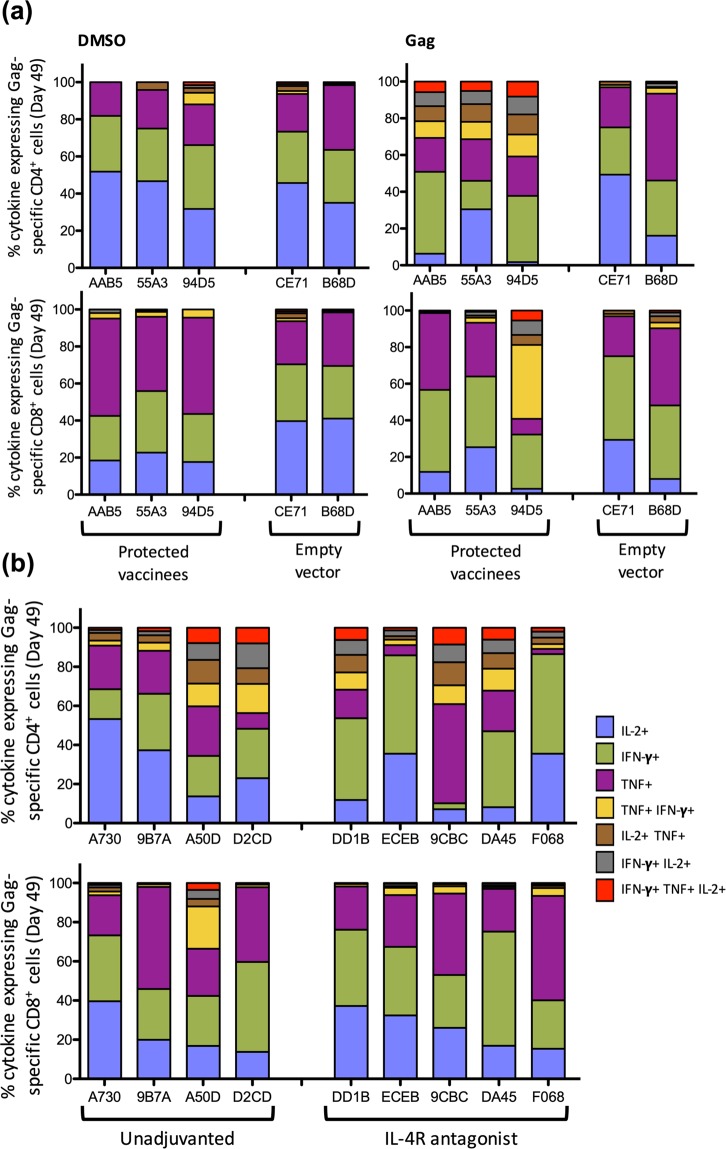
Poly-functionality of T cells in protected animals compared to controllers and progressors. The poly-functional cytokine expression profile of the systemic CD4^+^ T cells and CD8^+^ T cells following the first rMVA booster vaccination, was assessed by multi-colour intracellular cytokine staining. (**a**) The stacked bar charts represent the proportion of CD4^+^ T cells (top panel) and CD8^+^ T cells (bottom panel) from protected animals (AAB5, 55A3, 94D5) and empty vector vaccinated macaques (CE71, B68D) expressing a single cytokine (IFN-γ, TNF or IL-2 only), or expressing combinations of TNF/IFN-γ, IL-2/ TNF, IFN-γ/IL-2 and IFN-γ/ TNF/IL-2, are represented as stacked bar charts, following 15 mer overlapping SIV Gag-specific peptide stimulation, compared to DMSO stimulation. (**b**) The cytokine expression profile of the remaining macaques, in their respective groups, is also presented as stacked bar charts. Please see Supplementary Tables 1, 2 for absolute T cell counts.

The three vaccinees with undetectable viral loads showed highly poly-functional Gag-specific systemic CD4^+^ T cells following the first rMVA booster vaccination (Day 49), compared to the empty vector controls where the rare background cytokines evoked by Gag stimulation resulted in a similar cytokine profile to DMSO stimulation (Fig. 6a). Interestingly, however, the Gag-specific CD4^+^ T cells from these 3 animals displayed a more impressive poly-functional profile compared to their respective Gag-specific CD8^+^ T cells (Fig. 6a). When the remaining 9 viremic vaccines were assessed, similar proportions of Gag-specific poly-functional CD4^+^ T cells were observed across the macaques in both vaccine groups (Fig. 6b). In contrast, no difference in the CD8^+^ T cell poly-functionality was observed between the two vaccination groups.

Furthermore, it was also observed that the overall cytotoxicity (measured by CD107A expression) of systemic Gag-specific CD4^+^ T cells was similar in the two vaccinated groups at day 49 post challenge (see Supplementary Fig. S6). However, by day 126 post challenge, IL-4R antagonist vaccinees expressed significantly higher proportions of cytotoxic CD4^+^ T cells compared to the unadjuvanted group (see Supplementary Fig. S6). Unlike CD4^+^ T cells, between the two vaccine groups, the cytotoxicity of CD8^+^ T cells did not change over time. At day 126 post challenge, all antagonist vaccinees displayed CD107A^+^ cytotoxic Gag-specific CD4^+^ T cells > 0.8%, whilst only 3 out of 6 macaques displayed this profile in the unadjuvanted vaccine group (see Supplementary Fig. S6). The IL-2, IFN-γ and TNF expression profiles in T cells also significantly changed over the course of the study, with peak IFN-γ expression by CD4^+^ and CD8^+^ T cells being detected at 14 and 70 days post challenge respectively. In contrast, TNF expression by both CD4^+^ and CD8^+^ T cells peaked at 70 days post challenge and was maintained, whilst IL-2 expression was found to wane overtime (see Supplementary Fig. S7).

### Protection was associated with IL-2 expression by rectal and cervico-vaginal cytotoxic CD4^+^ T cells

**Figure 7:**
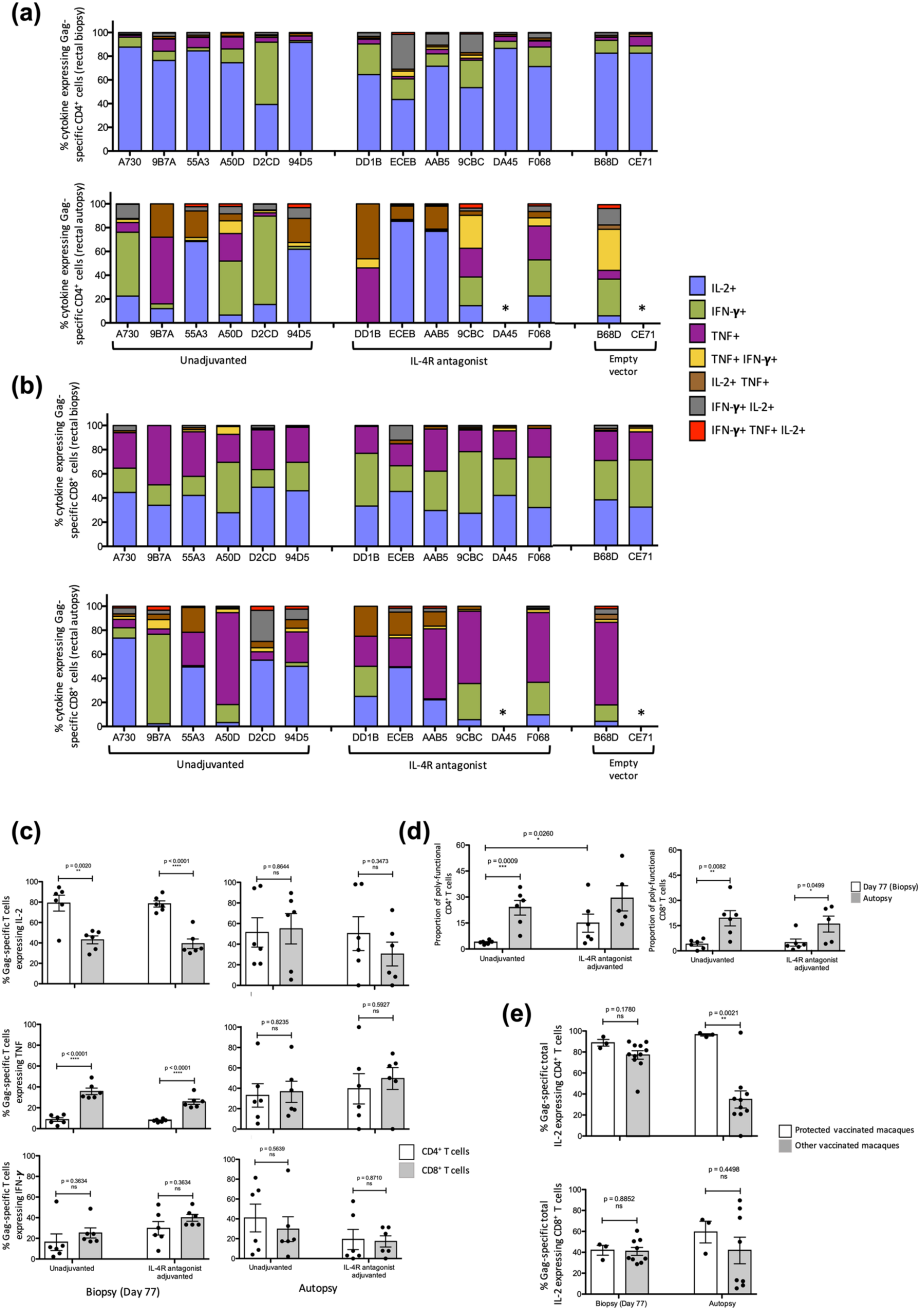
Evaluation of Gag-specific rectal CD4^+^ and CD8^+^ T cells at biopsy and autopsy. Rectal biopsies (day 77) and autopsies were performed and single cell suspensions were prepared from tissue samples, stimulated with 15 mer overlapping SIV Gag-specific peptide pool and their cytokine expression profiles were measured by multi-colour intracellular cytokine staining. Proportion of cytokine expressing (**a**) CD4^+^ T cells and (**b**) CD8^+^ T cells from biopsy (top panel) and autopsy (bottom panel) expressing the three cytokines of interest, IFN-γ, TNF, IL-2 individually or in a combination are presented as stacked bar charts as in Fig. 6. Rectal sample could not be obtained from DA45 and CE71 at autopsy and are hence labelled as “*”. Please see Supplementary Tables 3, 4 for absolute T cell counts. (**c**) The total IL-2 (top panel), TNF (middle panel) and IFN-γ (bottom panel) was also measured at both biopsy (left) and autopsy (right) by CD4^+^ and CD8^+^ T cells in both unadjuvanted and IL-4R antagonist vaccine groups. (**d**) The extent of CD4^+^ T cell (left) and CD8^+^ T cell (right) poly-functionality was also determined between the two vaccinated groups. (**e**) IL-2 expression by CD4^+^ T cells (top panel) and CD8^+^ T cells (bottom panel) from the three protected animals (AAB5, 55A3 and 94D5) were also compared to the remaining macaques at both biopsy and autopsy. Unpaired student’s t tests were performed to assess statistical significance.

**Figure 8:**
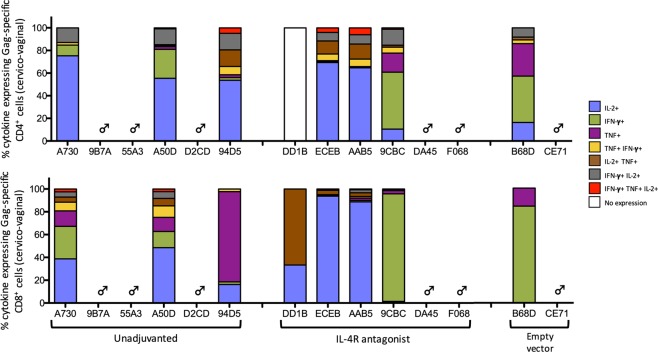
Evaluation of Gag-specific cervico-vaginal CD4^+^ and CD8^+^ T cells at autopsy. Cervico-vaginal autopsies were performed on all female macaques and single cell suspension were prepared from the isolated tissue samples, stimulated with 15 mer over-lapping SIV Gag-specific peptide pool and their cytokine expression profiles were measured by multi-colour intracellular cytokine staining. The stacked bar charts represent the proportion of cytokine expressing CD4^+^ T cells (top panel) and CD8^+^ T cells (bottom panel) expressing the three cytokines of interest as in Fig. 6. Please see Supplementary Table 5 for absolute T cell counts.

Cervico-vaginal and rectal tissues were also harvested to evaluate Gag-specific mucosal CD4^+^ and CD8^+^ T cell immunity. Rectal biopsies (Fig. 7a,b) from all 14 macaques were collected at Day 77 (7 days post second rMVA booster) and from 12 animals at autopsy. Cervico-vaginal (Fig. 8) tissue samples were obtained from the 8 (female) macaques at autopsy. Tissue samples were processed to obtain lymphocytes which were analysed by flow cytometry for SIV_mac239/251_ Gag-specific cytokine expression. Lymphocytes harvested from rectal biopsies revealed that in all macaques (Day 77), IL-2 was the predominant cytokine expressed by Gag-specific CD4^+^ T cells by both vaccine groups but was less commonly expressed by Gag-specific CD8^+^ T cells (p = 0.002 & <0.0001 respectively). In contrast, significantly higher TNF expression was more often detected in CD8^+^ than CD4^+^ T cells (p < 0.0001 for both vaccine groups) (Fig. 7a–c). However, similar IFN-γ expression profiles were detected in both CD4^+^ and CD8^+^ T cells. Post SIV challenge, at autopsy, no significant differences in the cytokine expression profiles were detected in CD4^+^ or CD8^+^ T cells (Fig. 7c).

Interestingly, the macaques in the IL-4R antagonist vaccine group displayed an overall higher proportion of poly-functional rectal CD4^+^ T cells at biopsy, compared to the macaques administered the unadjuvanted vaccine (p = 0.026) (Fig. 7d). CD4^+^ T cell poly-functionality was significantly higher at autopsy than biopsy among the unadjuvanted vaccinated macaques (p = 0.0009), but not the antagonist vaccinated macaques. CD8^+^ T cell poly-functionality, however, was significantly elevated at autopsy among all vaccinated macaques. It is noteworthy that early IL-2 dominant CD4^+^ T cell profiles in macaques 55A3, 94D5, DD1B, ECEB, and AAB5 was maintained till autopsy (Fig. 7a). Moreover, similar CD8^+^ T profile was maintained in A730, 55A3, D2CD, 94D5, DD1B, ECEB and AAB5 (Fig. 7b). The remaining macaques including F068, 9CBC, B68D, A50D and 9B7A, did not maintain the IL-2 predominant CD8^+^ T cell profile at autopsy. Furthermore, it was interesting that although CD4^+^ T cells from 9CBC, ECEB and AAB5 from the IL-4R antagonist vaccine group displayed a closely matched IL-2 dominant poly-functional profile at biopsy, at autopsy, only ECEB and AAB5 maintained this profile (Fig. 7a). In the 3 macaques that were completely protected, high IL-2 expression by rectal CD4^+^ T cells was maintained until autopsy and was significantly higher compared to the other vaccinated macaques (p = 0.0021) (Fig. 7e). However, in these animals no significant differences in the IL-2 expression by rectal CD8^+^ T cells were detected at biopsy and autopsy.

**Figure 9:**
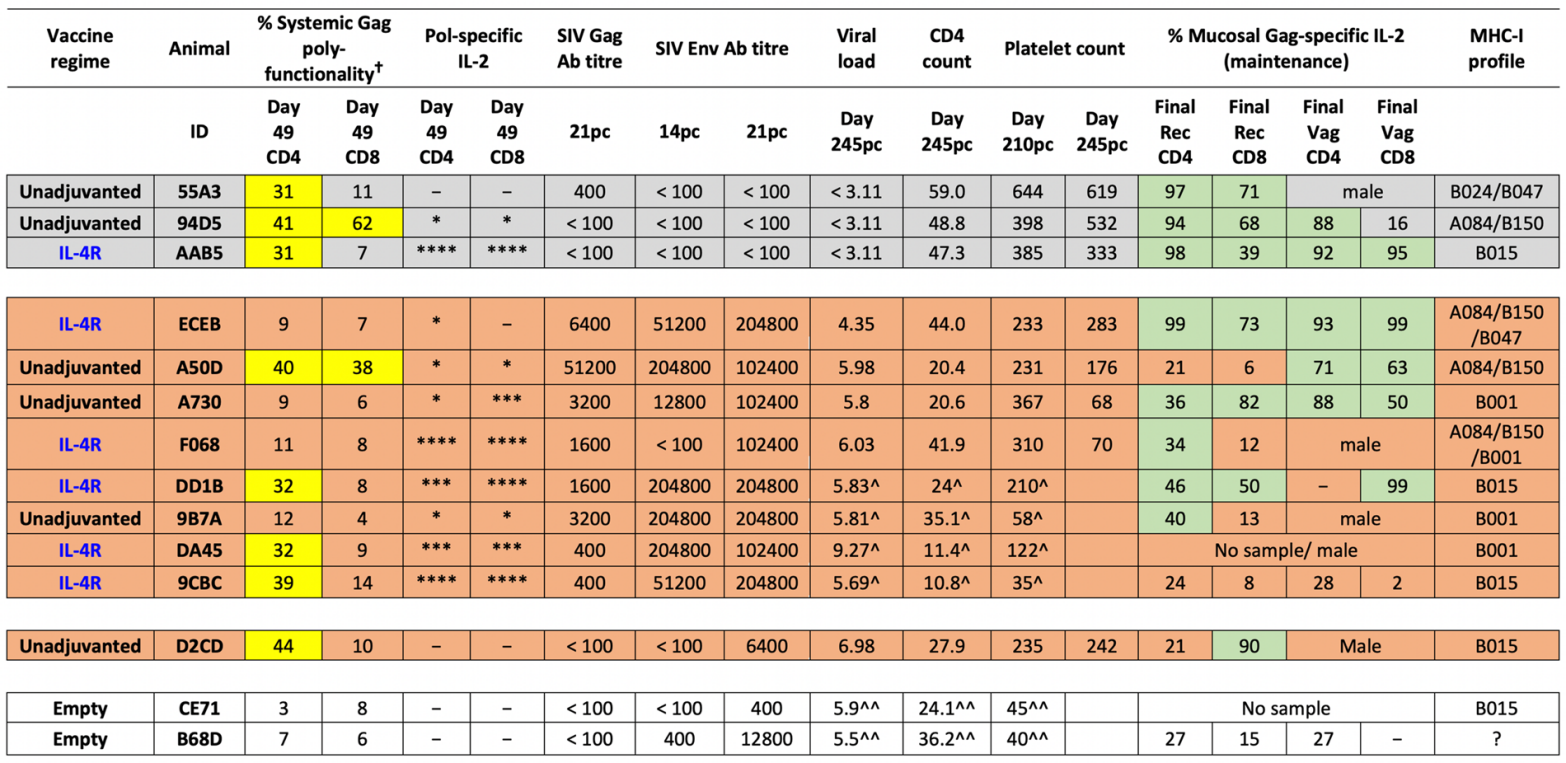
Trial summary. The findings from this trial are summarised in a colour coded table. Grey represents fully protected vaccinees, orange represents vaccinees eliciting effective T cell and/or humoral immunity, with some showing partial protection, and clear represents unprotected vaccinees with no effective humoral and/or T cell immunity. Yellow boxes represent systemic poly-functionality >30% and green represents mucosal poly-functionality >30%. Animals 9CBC and DA45 were euthanised on days 126 pc and 154 pc respectively, and 9B7A and DD1B on day 210 pc, all prior to the endpoint of the trial and are indicated as “^”. Unprotected animals B68D and CE71 were euthanised on days 126 and 156 respectively, and are indicated as “^^”. The percentage Gag-specific polyfunctionality (†) column represents the percentage of cells expressing combinations of IFN-γ, TNF and IL-2. Pol-specific IL-2 cell counts are presented using an asterisk system (*)where *<0.2; **0.2–0.4; ***0.4–0.6 and ****>0.6.

When poly-functional Gag-specific cytokine profiles in cervico-vaginal CD4^+^ and CD8^+^ T cells were assessed, IL-2 was once again identified as the predominant cytokine expressed by most vaccinees, either singly or in combination with TNF (Fig. 8). Poly-functional cytokine profiles of empty vector control animals were almost identical at the CD4^+^ and CD8^+^ T cell level, comprising of little-to-no IL-2 expression, with respective animals displaying a comparable profile in the rectal tissue (Fig. 9). Furthermore, similar to the systemic compartment, overall the rectal and cervico-vaginal CD4^+^ T cells were also found to be more cytotoxic compared to CD8^+^ T cells. However, unlike systemic compartment where the T cell cytokine profile progressed predominantly towards a TNF profile, mucosal T cells predominantly expressed IL-2.

## Discussion

In this study, albeit the relatively small sample size, intranasal rFPV prime followed by intramuscular rMVA booster vaccine strategy was able to induce protective immunity against a high dose pathogenic rectal SIV_mac251_ challenge in a subset of animals. Induction of effective mucosal immunity in the rectal and cervico-vaginal mucosae was one of the highlights of this study, which was not entirely surprising as we have previously shown that i.n. rFPV vaccination can induce robust immunity at the local and distal mucosae (specifically gut, rectal and genital mucosae)^27,39,43^. In this trial, the level of protection observed was multi-factorial. The complete protection (no detectable viral load) in the 3 protected vaccinees (94D5, 55A3 and AAB5) was associated with IL-2 driven Gag-specific poly-functional cytotoxic mucosal CD4^+^ T cells, complemented by Gag-specific poly-functional systemic CD4^+^ and mucosal CD8^+^ T cell immunity, but not humoral immunity (Fig. 9). Interestingly, despite the presence of poly-functional systemic CD4^+^ T cells equally in protected and some partially protected or non-protected macaques, if animals did not mount a strong IL-2 driven Gag-specific mucosal immune responses (eg: A50D, DD1B, 9CBC, D2CD) animals were not fully protected. Similarly, absence of poly-functional systemic CD4^+^ T cells and presence of robust cervico-vaginal and rectal CD4^+^ T cell responses (eg: ECEB, A730) also did not confer full protection. These findings elucidated that a balanced mucosal and systemic CD4^+^ T cell immunity was required for effective viral control (Fig. 9).

Our observations also suggested that if animals were unable to mount an effective CD4 response, animals not only required Gag or Pol-specific systemic CD8 T cell immunity, but also humoral immunity for effective control of infection (Fig. 9). Interestingly, macaque D2CD, although high viral load, did not succumb to disease, most likely due to the maintenance of poly-functional mucosal CD8^+^ T cells and systemic CD4^+^ T cells, along with high platelet counts. In the context of female animals, not only rectal (the site of the challenge in this trial), but also effective vaginal Gag-specific CD4 immunity was also appeared to play a role in protection (94D5, AAB5 vs A50D, DD1B) (Fig. 9). Overall, our trial data revealed that induction of sustained IL-2 driven Gag-specific mucosal cytotoxic CD4^+^ T cell immunity, was a key determinant of immune hierarchy and level of protection.

Complexities of understanding the exact mechanisms by which HIV evades the host immune system and also different HIV vaccination trials eliciting different immune parameters associated with protection, have compounded the design of an efficacious vaccine. A body of evidence indicates that cytotoxic CD4^+^ T cell mediated immunity is important for control of many viral infections, including HIV^57–59^. Notably, antigen-specific CD4^+^ T cells capable of expressing cytotoxic markers including, T cell intracellular antigen 1, granzymes A and B, CD161 (NKRP-1), CD244 (C1. 7/2B4), perforin, and GMP-17/TIA-1 along with IFN-γ, have been reported in HIV elite controllers^60,61^. Interestingly, macaques that were depleted of their CD8^+^ T cells have shown effective control SIV replication and viremia^62,63^ and elevated Gag- and Env- specific humoral responses, with circulating CD45RA^−^ CD28^+^ CD95^+^ CCR7^−^ granzyme B^+^ SIV-specific CD4^+^ T cells being associated with this protection^62^. Moreover, CD4^+^ T cells from HIV infected individuals, although low in frequency, have also displayed increased cytotoxicity compared to uninfected individuals^64,65^, suggesting that long-term HIV disease status is significantly dependent upon the level of cytotoxic CD4^+^ T cell immunity in HIV patients^66,67^. These findings are highly consistent with our unexpected findings where mucosal and systemic cytotoxic CD4^+^ T cells were mainly associated with complete protection. However, a CD4 depletion study may help further establish the role of cytotoxic CD4 T cells in SIV control, and this warrants further investigation, which is a caveat in this study.

Although IL-4R antagonist adjuvanted vaccine was not superior at controlling SIV viremia, it enhanced certain immune parameters compared to the unadjuvanted vaccine. Specifically, the IL-4R antagonist adjuvanted vaccine was able to induce elevated mucosal CD4^+^ T cell responses immediately following rMVA booster and also increased breadth of systemic T cell immunity, resulting in significantly elevated (~1–1.5 fold higher) Pol-specific IL-2 expression by both CD4^+^ and CD8^+^ T cells. Furthermore, despite being outbreed, the majority of the vaccinated macaques showed uniform and rapid Env-specific antibody responses at day 14 to 21 post SIV_mac251_ challenge (except for the three fully protected animals, where no virus was observed). The unique differences observed between adjuvanted versus unadjuvanted vaccines were not entirely surprising as our murine studies have shown that i.n. delivery of IL-4R antagonist adjuvanted vaccine can recruit uniquely different dendritic cell subsets to the lung mucosae, responsible for induction of high quality T and B-cell immunity^38^. Moreover, we have recently shown that transient inhibition of STAT6 signalling and promoting IL-13 signal via alternate pathway can promote effective B cell differentiation^30,68^.

The second rMVA booster did not further improve the initial T cell responses, but unexpected uniform/rapid anamnestic Env-specific antibody responses were observed following a single rFPV prime that contained *env* antigens, 14 days post SIV_mac251_ challenge. This most likely indicated that the second i.m. rMVA booster could have enhanced vector-specific T cell immunity, and not immunity to the encoded antigens^69^. Moreover, now knowing that the immune cells have a limited lifespan *in vivo*, and repeated antigenic stimulation can render T cells exhausted and less differentiated, resulting in poor memory T cells^70–74^. Therefore, in the context of viral vector-based vaccination, ‘less could be more’^71,72^. Whether an additional protein booster may prove more efficacious in our system to enhance antibody immunity warrants further investigation. Thus, a head-to-head comparison of the two vaccines with and without an additional Env protein booster, similar to RV144 trial, may help further differentiate the efficacy of the two vaccines.

Collectively, our findings further substantiate rFPV as a promising intranasal delivery vector. The level of protection following mucosal pathogenic challenge was associated with an immune ‘hierarchy’, where cytotoxic CD4^+^ T cell mediated mucosal and systemic immunity correlated strongly with ‘complete protection’. In the absence of an effective mucosal and systemic CD4^+^ T cell immunity, our vaccination regimens resulted in the induction of humoral and CD8 T cell responses as a possible ‘rescue’ mechanism in order to ensure some level of protection. Co-expression of the IL-4R antagonist, together with HIV antigens, improved the breath of immune responses and the responsiveness of both CD4^+^ and CD8^+^ T cells, making it a worthy vaccine strategy for further investigation.

## Materials and Methods

### Animals

Fourteen healthy juvenile pigtailed macaques (*Macaca nemestrina*) were used for the study. Macaques were randomly grouped such that six received the parental unadjuvanted vaccine (FPV/MVA) with both vectors expressing SIVmac_239_ *gag/pol/env*, six received IL-4R antagonist FPV/MVA vaccines expressing SIVmac_239_ *gag/pol/env* and an IL-4R antagonist. The remaining two macaques received FPV/MVA vectors not expressing SIV antigens nor adjuvants vaccine (Table 1). Rationale behind using only two macaques in this group was that our previous studies have consistently shown that vaccination with empty vector does not induce any SIV specific immunity^37,54^. Macaques were MHC-I typed (kindly performed by Dr. David O’Connor and Roger Wiseman, University of Wisconsin^54^) and a subset of 4 macaques were Mane-A1*084 positive, which restricts the SIV Gag CTL epitope KP9 epitope in Gag and could be studied for CD8 T cell responses using a Mane-A1*084/KP9 tetramer as previously described^51–53^.

### Ethics statement

All the animals used in this study were used under the Australian Animal Health Laboratory Animal Ethics Committee (AAHL AEC) and the University of Melbourne’s Animal Ethics Committee approved guidelines. Juvenile *Macaca nemestrina* free of simian immunodeficiency virus, simian retrovirus type D and simian T cell leukaemia virus infections, were obtained from the Australian macaque breeding facility and housed under physical containment level 3 conditions. Animals were housed in large nonhuman primate cages (3 m × 2 m × 1 m) and cared for as per the Australian National Health and Medical Research Council guidelines and protocol approved by the relevant institutional Animal Ethics Committees. All animals received standard primate feed and free access to water, maintained in a 12 hr light/12 hr dark cycle and monitored daily by experienced animal technicians. All procedures, including immunisations and tissue harvest, were performed by experienced staff. A sedative, Ketamine (10 mg/kg intramuscular), was administered prior to performing any procedures. At the end of the trial period, macaques were ethically euthanized with Phenobarbitone.

### Constructions of vaccines

The FPV-SIV_mac239_ *gag/pol/env* parent vaccine was kindly provided by Dr David Boyle, and has successfully been used in previous NHP trials^75^. The macaque gene for the IL-4 antagonist (IL-4C123) were deduced and synthesized based on the rhesus macaque genome sequence (GeneBank NCBI Reference sequence: NM_001032904) by comparison to the human and mouse genes (see Supplementary Fig. S8). FPV-SIV_mac239_ *gag/pol/env* parent virus, co-expressing the macaque IL-4C123 gene driven by a late promoter was constructed as described previously^47,76^. Similarly, MVA-SIV_mac239_ *gag/pol* parent vaccine and the MVA-SIV_mac239_ *gag/pol* co-expressing macaque IL-4C123 gene were constructed (see Supplementary Fig. S8)^77^. The vaccine constructs were plaque purified and validated for insertion and expression of the macaque IL-4C123 and SIV genes by PCR and western blotting using methods described previously^75,78^.

### Immunisation and SIV challenge

Macaques were primed with 2 × 10^8^ pfu of the respective rFPV vaccine both intranasally using LMA® MAD™ intranasal mucosal atomization device (Teleflex /USA), and directly to the lung using a tube placed into the trachea laryngoscopically. Four weeks later, macaques were boosted intramuscularly twice with the respective rMVA vaccines (2 × 10^8^ pfu initially and a second dose of 1 × 10^8^ pfu) four weeks apart as per timeline (see Supplementary Fig. S1 and Table 1). As the primary outcome was T cell based control of viremia, all animals were administered a single high dose (10^3^ TCID_50_) intrarectal SIV_mac251_ challenge at day 98. The SIV_mac251_ (SIV_mac239_ derived) stock was kindly generated by Dr. Ron Desrosiers and supplied by Dr Nancy Miller as previously^79^. Blood samples were collected at various time points to evaluate protective efficacy (see Supplementary Fig. S1). The animal behaviour, weight, injection site reactions, peripheral blood haematology and CD4^+^ T cell counts were monitored for the duration of the trial as previously^79^.

### Assessment of SIV infection

The copy number of SIV RNA per millilitre of plasma were measured on serial blood samples using quantitative reverse transcription-polymerase chain reaction (RT-PCR), as described previously^80,81^. Briefly, RNA was purified from plasma and reverse-transcribed. Quantification of viral RNA copies within plasma was accomplished using a TaqMan minor groove binding 5′ fluorescently labelled probe and a 3′ non-fluorecently labelled quencher (with the limit of detection set at 3.1 log_10_ copies/mL). To validate the results of selected samples with undetectable viral loads, RT-PCR was also kindly performed on pelleted virions from 1.0 mL of plasma at the National Cancer Institute by Dr Jeff Lifson and colleagues (limit of detection 1.5 log_10_ copies/mL) as described previously^82^.

### Detection of SIV_mac239/251_ Gag and Env (gp140) specific monoclonal IgG antibody titres

ELISAs were used to determine plasma IgG antibodies specific to SIV_mac239/251_ Gag and Env gp140 proteins (Immune Technology Corp)^47^. Briefly, 96-well ELISA plates (ThermoFisher Scientific) were coated with Gag or gp140 at 1 ng/well overnight. Plates were blocked with 5% skim milk, serial dilutions of plasma (1:400 to 1:102,400) added and incubated overnight at 4 °C. Next, HRP-conjugated anti-macaque IgG (HRP-1B3 – NIH Non-Human Primate reagent resource facility, University of Massachusetts Medical School), followed by peroxidase substrate, 2,2′-azino-bis (3-ethylbenzothiazoline-6-sulphonic acid) were added and the optical densities (OD) were read at 405 nm on a Tecan Infinite m200 Pro Spectrometer.

### Preparation of mucosal tissue for Intracellular cytokine staining (ICS)

Tissue samples were collected at Day 77 (7 days post second rMVA booster, rectal biopsies) and at autopsy (rectal and cerivo-vaginal tissues). All mucosal tissues samples were stored in RPMI-1640 (Sigma) supplemented with 25% foetal bovine serum until use the same day. Single cell suspensions were prepared for intracellular cytokine staining (ICS) as described previously^39,43,47^. Briefly, tissue samples were mechanically cut into small pieces and incubated in complete RPMI (10% FBS) containing 2 mg/mL collagenase (Sigma), 2.4 mg/mL dispase (Gibco-Invitrogen) and 5 Units/mL DNAse (Calbiochem La Jolla, CA), for 45 min at 37 °C, with regular vortexing every 10 min, digested tissue was passed through two layers of sterile gauze to remove debris, centrifuged and resuspended in erythrocyte lysis buffer (0.15 mM NH_4_Cl, 10 mM KHCO_3_, 0.1 mM Na_2_EDTA) for 2 min at room temperature. Cells were washed twice in complete RPMI and passed through a 100 μm cell strainer (Sigma) to remove any remaining debris. Finally, cells were centrifuged and resuspended in 0.5–1 mL of complete RPMI.

### Intracellular cytokine staining (ICS)

Expression of cytokines IFN-γ, TNF, IL-2 and IL-17A, by lymphocytes in fresh whole blood^81,83,84^ and mucosal samples^39,43,47^ were measured by flow cytometry as described previously. Briefly, 200 μL of whole blood was incubated at 37 °C with 1 μg/mL overlapping 15-mer Gag or Pol peptide sets (NIH AIDS Reagent Program), or dimethyl sulfoxide (DMSO - Sigma) or Staphyloccal Enteroxin B (SEB - Sigma), along with 1 μg/mL co-stimulatory antibodies anti-CD28 and anti-CD49d (BD-Biosciences) in the presence of 10 μg/mL Brefeldin A (Sigma) for 6 hr. Cells were surface stained with CD3-PB (clone SP34-2), CD4-PerCP (clone L200), CD8-BV650 (clone SK1) and CD107A-APC-H7 (clone H4A3) (all from BD-Biosciences) on ice for 30 min. Erythrocytes were lysed using FACS Lysing solution (BD Biosciences) and washed with PBS prior to permeabilizing using FACS Permeabilizing solution 2 (BD-Biosciences). Permeabilized cells were incubated with intracellular cytokine staining antibodies IFN-γ-APC (clone B27, Biolegend), IL-17A-PE (clone 64CAP17, eBiosciences), TNF-α-PE-Cy7 (clone mAB11) and IL-2-FITC (clone MQ1–17H12) (both from BD-Biosciences) on ice for 30 min prior to fixing with 0.5% paraformaldehyde, acquiring 2 × 10^6^ cells on a BD LSRFortessa and analysing data using FlowJo version 10.3 software.

Following mucosal single cell preparation, 4 × 10^6^ cells were stimulated as above, however, for 12 hr at 37 °C. Brefeldin A was added to the plate and incubated for a further 5 hr at 37 °C. Surface staining was performed as described previously and cells were fixed in fixation buffer (BD-Biosciences) and permeabilized using 1x pereabilizing buffer (eBiosciences) prior to staining with the intracellular cytokine antibodies, similar to whole blood. Finally, cells were fixed in 0.5% paraformaldehyde and 1 × 10^6^ were acquired on a BD LSRFortessa and data were analysed using FlowJo software.

### Tetramer staining

Immune responses to the immunodominant SIV Gag KP9 CD8^+^ T cell epitope in the Mane-A*084 macaques were assessed by tetramer staining as described previously^85^. Briefly, the Mane-A*084 polypeptide, a human β2-nicroglobulin and the KP9 peptide (GL Biochem) were complexed to form the KP9/Mane-A*10 tetramer. The tetramer complex was then conjugated to PE. Fresh whole blood (200 μL) was first stained with the KP9/Mane-A*A084 tetramer (1:200 dilution) for 30 min at room temperature, followed by counterstaining using CD3-PB and CD8-BV650 and subsequent red cell lysis. Cells were washed and fixed and data acquired on a BD LSRFortessa and analysed using FlowJo software.

## Statistics

For all T cell-based assays, SEM was calculated and *p*-values determined using two-tailed, two sample equal variance or unequal variance Student’s *t*-test to compare groups using GraphPad Prism Software 5.0 f. To determine endpoint titres (level of antibody in each sample), background absorbance readings were first determined across all ELISA plates and average was determined for endpoint titre calculation as described previously^47^. A plasma sample was considered as positive when the OD was 2 or 3 times that of the average background reading.

## Supplementary information

Supplementary Fig. S1–8; Supplementary Tables 1–5

## Data Availability

The authors declare that all data supporting the findings of this study are available with the paper and supplementary file.
