# Supplementary material for: Mucosal and systemic SIV-specific cytotoxic CD4+ T cell hierarchy in protection following intranasal/intramuscular recombinant pox-viral vaccination of pigtail macaques
Source: Sci Rep. 2019 Apr 5;9:5661. doi: 10.1038/s41598-019-41506-5 (PMC6450945; doi:10.1038/s41598-019-41506-5)
Supplement: Supplementary file 1 — Supplementary Fig. S1–8; Supplementary Tables 1–5 [file 41598_2019_41506_MOESM1_ESM.pdf]

Mucosal and systemic SIV-specific cytotoxic CD4$^+$ T cell hierarchy in protection following intranasal/intramuscular recombinant pox-viral vaccination of pigtail macaques

Running Title: IL-4R antagonist adjuvanted vaccine strategy

Mayank Khanna[1,4], Ronald J. Jackson[1], Sheilajen Alcantara[2], Thakshila H. Amarasena[2], Zheyi Li[1], Anthony D. Kelleher[3], Stephen J. Kent[2¶] and Charani Ranasinghe[1*¶]

[1]Molecular Mucosal Vaccine Immunology Group, Department of Immunology and Infectious Disease, The John Curtin School of Medical Research, The Australian National University, Canberra ACT 2601, Australia

[2]Department of Microbiology and Immunology, Peter Doherty Institute, University of Melbourne, Melbourne VIC 3010, Australia

[3]Immunovirology and Pathogenesis Program, Kirby Institute, University of New South Wales, Sydney NSW 2052, Australia

[4]Department of Microbiology, Immunology and Parasitology, Louisisana State University Health Sciences Center, New Orleans, LA 70112, USA (current).

¶CR and SK, contributed equally to this work

*Correspondence: Charani.Ranasinge@anu.edu.au; Telephone: +61 2 6125 4706

Supplementary Fig. S1

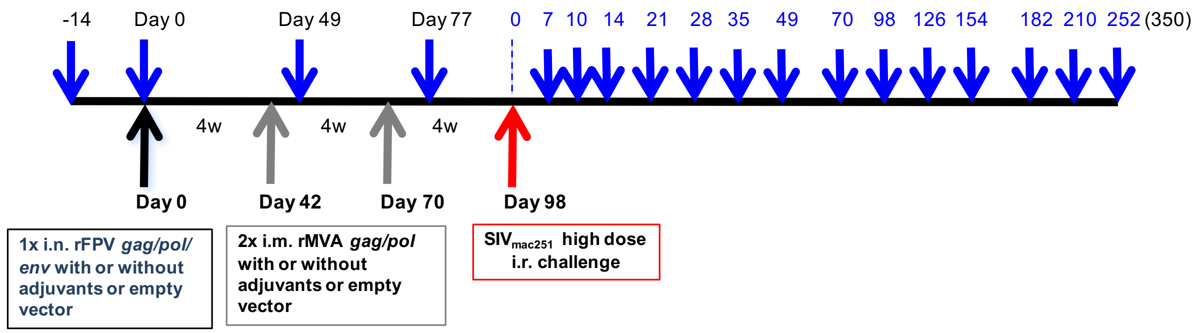

Figure S1: Sixteen macaques were i.n./i.m. prime-boost immunised four weeks apart, using $1x10^7$ PFU of the respective rFPV vaccines (black arrow) and $2x10^7$ and $1x10^7$ of the respective rMVA vaccines (grey arrows) as per indicated in **Table 1**. At day 98 (red arrow) all macaques were challenged intrarectally with a high dose SIV_{mac251} and were monitored for further 252 days. Blood samples were collected at time intervals indicated in blue arrows. Rectal biopsies were collected at day 77 and at autopsy. The cervico-vaginal tissues were only collected at autopsy.

Supplementary Fig. S2

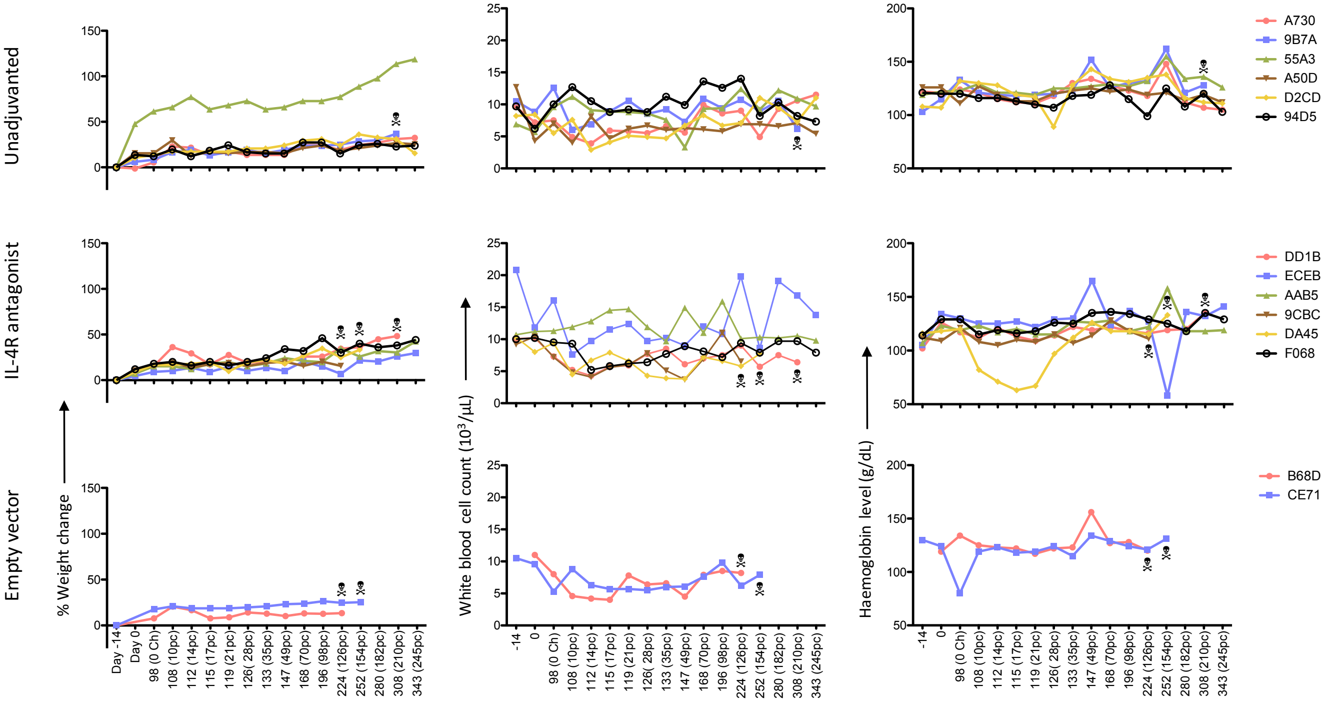

Figure S2: All fourteen macaques were weighed and bled at regular intervals as per timeline pre and post the high dose intrarectal SIV_{mac251} challenge. The weight change percentage (left panel) was determined using the respective weights at the beginning of the trial, as reference. Systemic white blood cell counts (middle panel) and the haemoglobin levels (right panel) were also determined. The macaques are grouped in their respective vaccine groups, with "☠" representing the time point when an animal was euthanized.

Supplementary Fig. S3

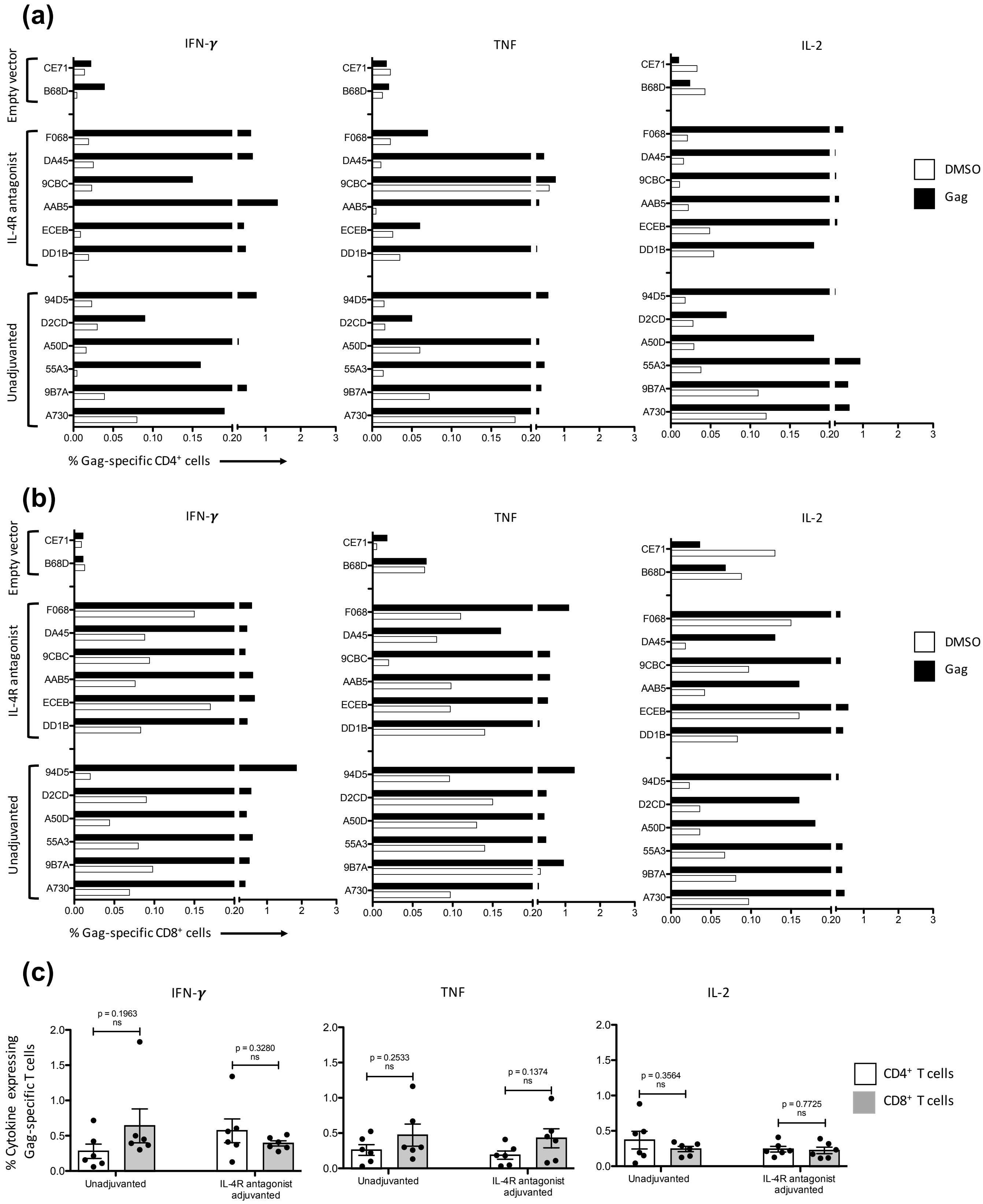

Figure S3: Freshly isolated lymphocytes from the whole blood of all fourteen animals from bleed at day 49 (after first rMVA booster), were stimulated with a 5 mer overlapping SIV Gag-specific peptide pool (black bars) and DMSO (white bars) and their cytokine expression measured by multi-colour intracellular cytokine staining. Bar charts show proportion of (a) $CD4^+$ T cells and (b) $CD8^+$ T cells expressing IFN-γ (left panel), TNF (middle panel) and IL-2 (right panel). A comparison between $CD4^+$ and $CD8^+$ T cells for their ability to express the three cytokines was also determined. (c) bar charts show proportion of $CD4^+$ (white bars) and $CD8^+$ (grey bars) T cells expressing IFN-γ (left panel), TNF (middle panel) and IL-2 (right panel). Unpaired student's t tests were performed to assess statistical significance.

Supplementary Fig. S4

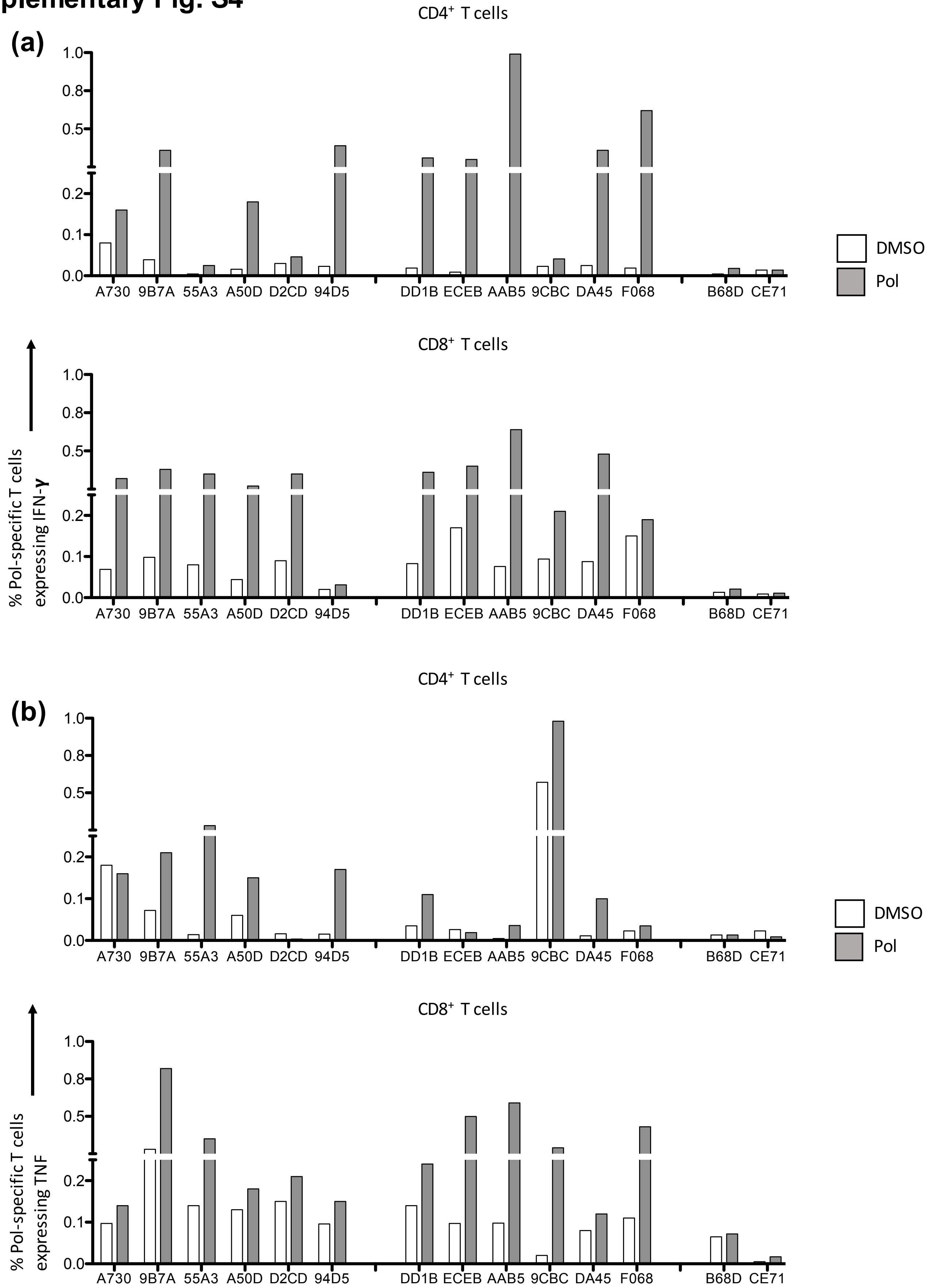

Figure S4: Freshly isolated lymphocytes from the whole blood of all fourteen animals from bleed at day 49 (after first rMVA booster), were stimulated with a 5 mer overlapping SIV Pol-specific peptide pool (grey bars) and DMSO (white bars) and their cytokine expression measured by multi-colour intracellular cytokine staining. Bar charts show proportion of (a) IFN-γ and (b) TNF by $CD4^+$ T cells (top panel) and $CD8^+$ T cells (bottom panel).

Supplementary Fig. S5

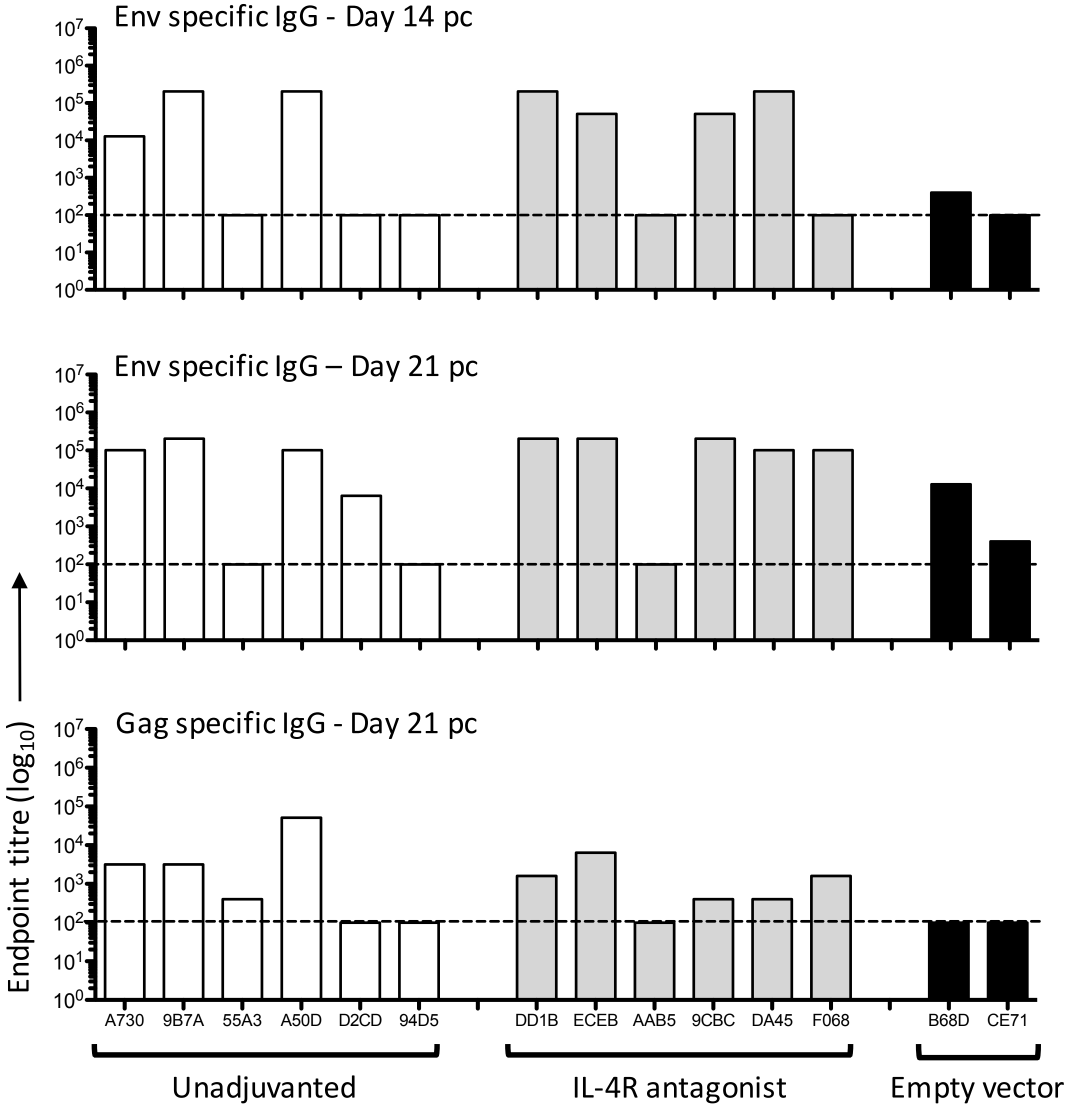

Figure S5: Env- and Gag-specific total IgG in the plasma of all fourteen macaques post SIV_{mac251} challenge were determined using ELISAs and used to calculate endpoint titres as described in methods section. Endpoint titres for Env-specific IgG was determined for days 14 (top panel) and 21 (middle panel) post challenge, whereas, similar titres for Gag-specific IgG was determined for day 21 post challenge (bottom panel).

Supplementary Fig. S6

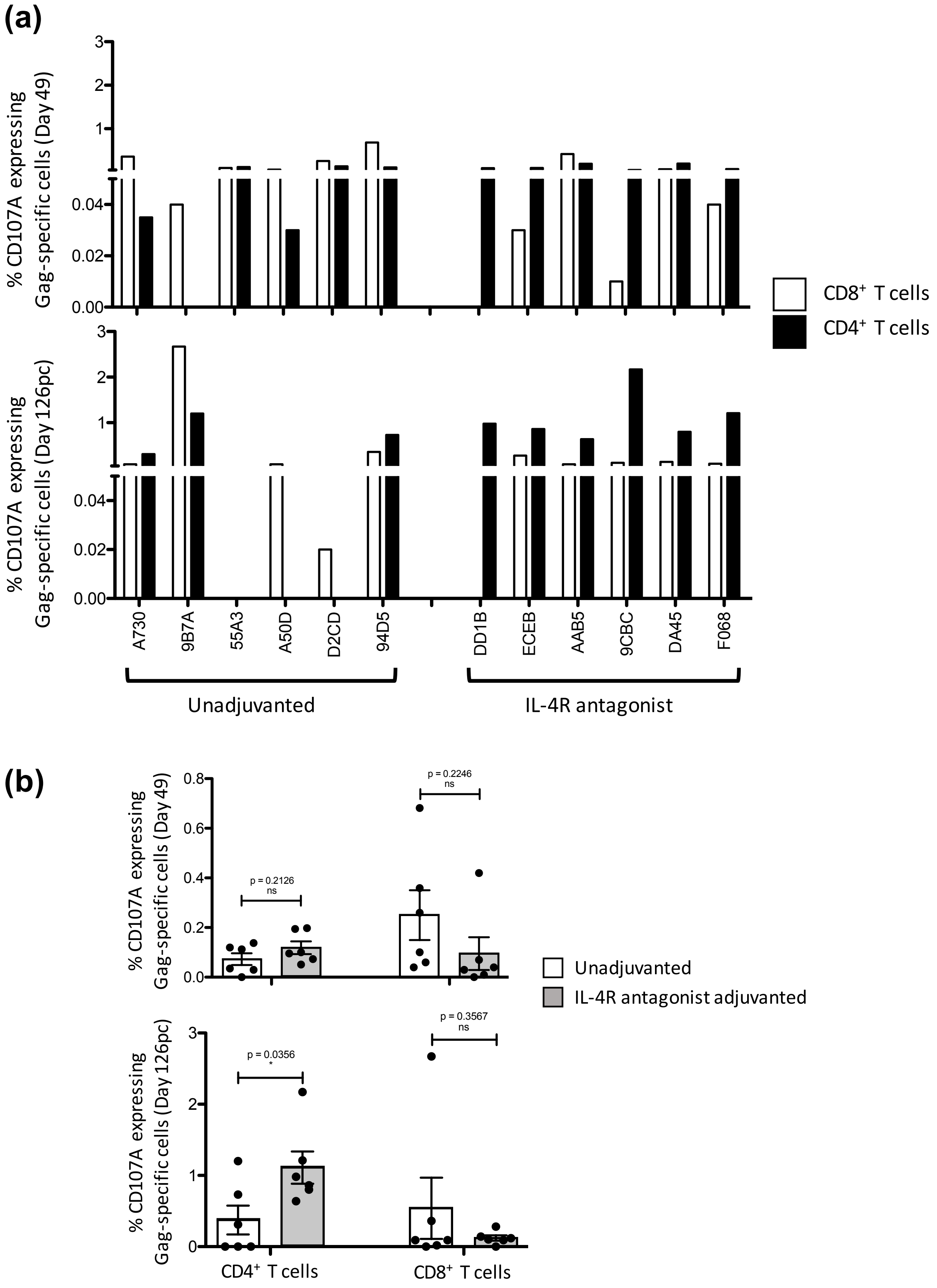

Figure S6: Freshly isolated lymphocytes from the whole blood of all vaccinees bleed at day 49 (after first rMVA booster) and day 126 post challenge, were stimulated with a 5 mer overlapping SIV Gag-specific peptide pool and the level of cytotoxicty of T cells determined by measuring CD107A expression by multi-colour fluorescence cytometry. (a) bar charts show proportion of CD107A expression by $CD8^+$ (white bars) and $CD4^+$ (black bars) T cells from samples at day 49 (top panel) and 126 post challenge (bottom panel). (b) a comparison between the cytotoxicity of the respective T cell populations in the two vaccine groups was also performed. The CD107A expression by T cells from the unadjuvanted (white bars) and IL-4R antagonist adjuvanted (grey bars) at day 49 (top panel) and day 126 post challenge (bottom panel) is presented as bar graphs. Unpaired student’s t tests were performed to assess statistical significance.

Supplementary Fig. S7

(a)

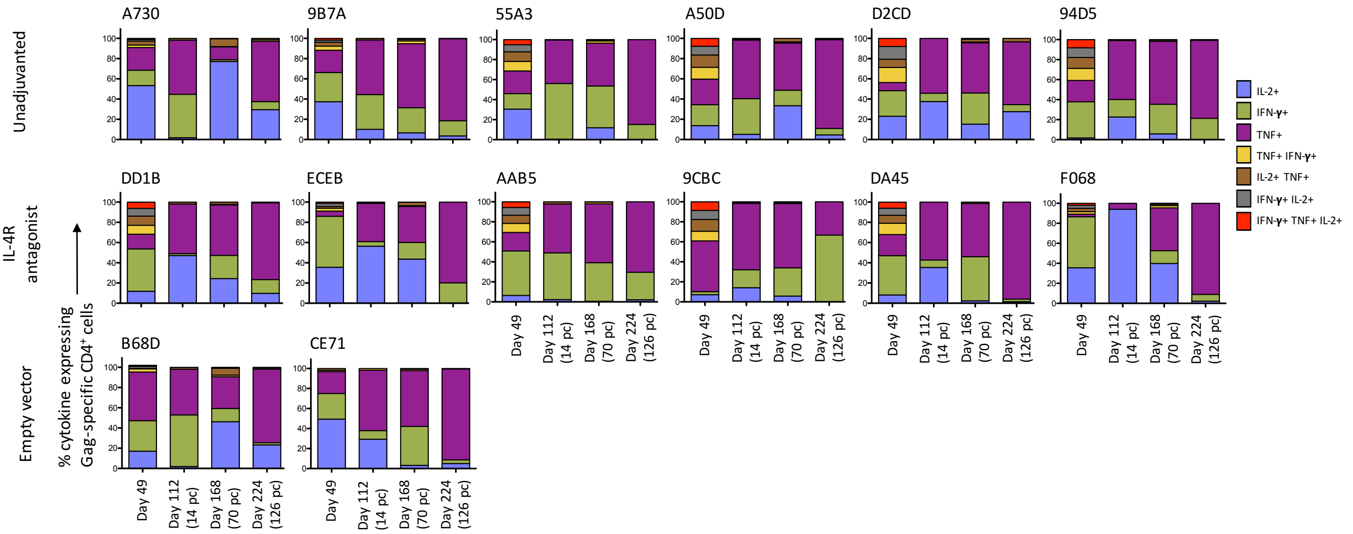

(b)

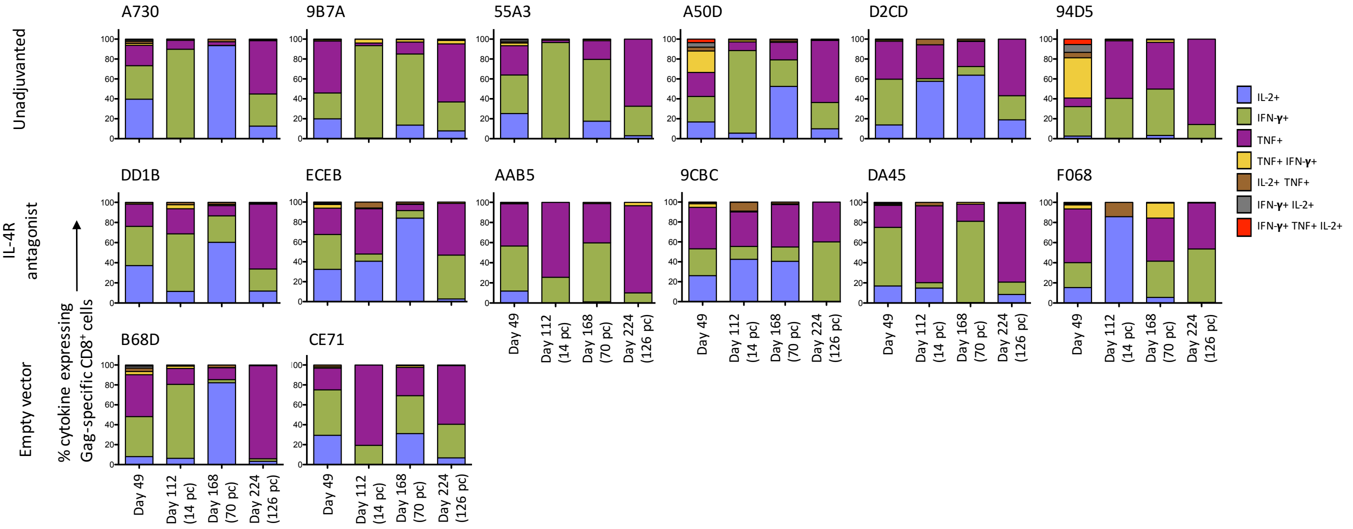

Figure S7: The poly-functional cytokine expression profile of the systemic CD4$^+$ T cells and CD8$^+$ T cells was assessed by multi-colour intracellular cytokine staining at various time points during the trial. The stacked bar charts represent the proportion of (a) CD4$^+$ T cells and (b) CD8$^+$ T cells from all fourteen macaques expressing a single cytokine (IFN-γ, TNF or IL-2 only), or a combinations of TNF/IFN-γ, IL-2/ TNF, IFN-γ/IL-2 and IFN-γ/ TNF/IL-2, are represented.

Supplementary Fig. S8

(a) Recombinant FPV construction:

The recombinant FPV containing SIV *gag* and *pol* genes in the "F" site (FPV089) was constructed by Coupar et al 2006 (Vaccine 9:1378) (Boyle et al 2004, BioTechniques 37:104). This FPV was further modified to include the macaque mutant IL-4C123 gene (IL-4R antagonist). The IL-4C123 is a synthetic C-terminal deletion of IL-4 gene deleted codons encoding for residues 124 (essential Y124) and downstream amino acids. The IL-4C123 gene was deduced from the macaque genomic DNA sequence on Genbank comparing to the known exon maps of the human gene. Gene sequence was synthesised by Genscript to custom order, as were the later optimised SIV*env* and SIV*gagpol* cassettes.

The synthetic IL-4C123 was separately ligated into the BamHI-HindIII in the MCS of FPV plasmid vector pAF09 (Heine & Boyle 1993, Archives of Virology 131: 277). Recombinant FPV encoding IL-4C123 was constructed by lipofectamine 2000 mediated transfection of FPV089 infected chicken embryonic skin cells (CES) as recommended by the manufacturer. Recombinant FPVs were isolated using mycophenolic acid/xanthine selection as described by Heine & Boyle and confirmed by beta-galactosidase expression (lacZ/X-gal).

The recombinant FPV089 and FPV089-IL-4C123 were further modified by insertion of the synthetic SIV*env* gene into the REV insertion site (Boyle et al 2004. The synthetic SIV*env* gene was ligated into the plasmid pFPVREV (Jackson, unpublished) along with the GFP-BSD cassette. pFPVREV is a synthetic DNA sequence with the remnant reticuloendotheliosis virus DNA sequences deleted (including the cryptic FPV202), generating an intergenic insertion site between FPV201 and FPV203 ligated into the vector pUC57NotI (Blue-white vector, single NotI site in frame with lacZ alpha, Jackson, unpublished).

Viruses FPV089-SIVenv, FPV089-SIVenv-IL-4C123 were constructed by homologous recombination transfection as above, however selecting for blasticidin S resistance and GFP+ plaques. Viruses were confirmed by PCR gene specific primers to confirm gene insertions.

Supplementary Figure 8a:

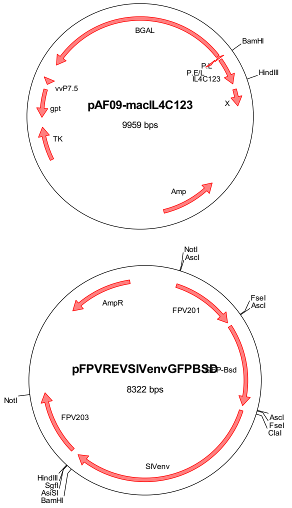

(b) Recombinant MVA construction:

MVA was plaque purified on CES cells and amplified for this study. Recombinant MVA expressing the macaque IL-4C123 was constructed using a unique plasmid pMVAdIII (Jackson unpublished). pMVAdIII contains a synthetic sequence with an intergenic insertions site located within the natural Deletion-III site of MVA. The immune-regulatory genes were inserted along with the bacterial GUS gene (Jackson et al 1996, J Gen Virol, 77: 1569) as a colour marker using the transient dominant selection method (Falkner & Moss 1990, J. Virol. 64:3108 ; Jackson & Bults 1992 J Gen Virol. 73: 3241).

The recombinant viruses were isolated using the transient dominant selection for blasticidin S resistance and GFP expression. The final recombinant viruses did not express either GFP or BSD as the GFP-BSD cassette is deleted during the second recombination event, however retain the Gus (X-Gluc, blue) and immunomodulatory genes.

The optimised SIV*gagpol* cassette was synthesised to order by Genscript. Both the *gag* and *pol* genes were optimised for expression in macaques based upon the species codon usage table. However, the overlapping sequences of the *gag* and *pol* open-reading frames was not modified as this contains secondary RNA folding sequences which directs frame-shift slippage translation at the polyT and expression of gag-pol fusion proteins.

Recombinant MVAwt and MVA-IL-4C123 co-expressing SIV*gagpol* were then isolated by plasmid transfection using Lipofectamine 2000 and virus infection. The plasmid vector pMVA-F (Jackson, unpublished) contains a synthetic sequence with an intergenic insertion site generated between ORFs F7L (MVA036) and F8L (MVA037). The synthetic SIV*gagpol* cassette was inserted along with the GFP-BSD cassette into the pMVA-F vector.

Recombinant viruses MVA-SIVgagpol and MVA-SIVgagpol-IL-4C123 were isolated using permanent dominant selection for blasticidin S resistance and GFP expression.

Supplementary Figure 8b:

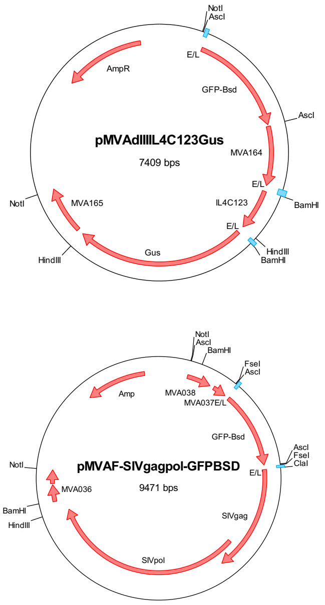

Supplementary Table 1: Absolute Gag-specific systemic CD4+ T cell counts (per 10^6) corresponding to Figures 6a#

	Animal ID	IL-2+	IFN-γ+	TNF+	TNF+ IFN-γ+	IL-2+ TNF+	IFN-γ+ IL-2+	IFN-γ+ TNF+ IL-2+
Protected vaccinees	AAB5	1208	8483	3518	1730	1565	1456	1099
	55A3	4589	2337	3422	1431	1450	1072	778
	94D5	259	5410	3207	1798	1636	1458	1231
Empty vector	CE71	913	474	405	23	35	0	0
	B68D	679	1266	1996	133	19	84	42
Unadjuvanted	A730	5639	1614	2362	266	416	166	116
	9B7A	5123	3966	3019	578	514	289	241
	A50D	941	1428	1742	801	836	592	540
	D2CD	461	507	161	300	161	254	161
IL-4R antagonist	DD1B	981	3480	1211	731	750	634	519
	ECEB	2471	3508	358	198	123	207	94
	9CBC	837	362	5998	1132	1403	1064	1018
	DA45	1013	4856	2600	1396	994	860	765
	F068	3800	5442	281	270	352	328	211

#Please note that the table layout corresponds to the figure layout.

Supplementary Table 2: Absolute Gag-specific systemic CD8+ T cell counts (per 10^6) corresponding to Figures 6b#

	Animal ID	IL-2+	IFN-γ+	TNF+	TNF+ IFN-γ+	IL-2+ TNF+	IFN-γ+ IL-2+	IFN-γ+ TNF+ IL-2+
**Protected vaccinees**	**AAB5**	1538	5795	5438	111	45	22	0
	**55A3**	3436	5251	3994	365	172	264	101
	**94D5**	669	7603	2194	10373	1394	2026	1394
**Empty vector**	**CE71**	543	844	405	23	35	0	0
	**B68D**	338	1692	1777	133	145	85	48
**Unadjuvanted**	**A730**	4167	3529	2153	199	219	140	100
	**9B7A**	3556	4630	9307	239	72	48	0
	**A50D**	1436	2178	2056	1837	328	389	304
	**D2CD**	1579	5277	4372	154	39	39	19
**IL-4R antagonist**	**DD1B**	3943	4132	2337	142	24	24	0
	**ECEB**	5267	5682	4287	619	129	194	65
	**9CBC**	3083	3189	4921	436	138	64	0
	**DA45**	1197	4130	1549	70	47	70	23
	**F068**	2945	4747	10221	774	219	162	104

#Please note that the table layout corresponds to the figure layout.

Supplementary Table 3: Absolute Gag-specific rectal $CD4^+$ T cell counts (per 10^6) corresponding to Figure 7a[#]

		Animal ID	IL-2+	IFN-γ+	TNF+	TNF+ IFN-γ+	IL-2+ TNF+	IFN-γ+ IL-2+	IFN-γ+ TNF+ IL-2+
Rectal biopsy	Unadjuvanted	A730	326018	30899	5969	527	3160	5267	0
		9B7A	106821	10811	14414	515	2574	4118	515
		55A3	150142	4843	15385	855	2564	3704	285
		A50D	109603	17119	14823	1044	3967	626	0
		D2CD	113856	152152	11530	3706	2882	5353	206
		94D5	290350	4371	12609	336	6725	2354	0
	IL-4R antagonist	DD1B	124141	49860	7632	1781	2798	5851	509
		ECEB	159321	63223	7587	16618	6142	107298	5780
		AAB5	98106	14281	5123	2949	2018	13039	1552
		9CBC	163197	70421	5030	7964	6427	48065	3773
		DA45	135235	9315	6440	460	2185	2415	115
		F068	147162	34061	10480	1310	3930	8734	873
	Empty vector	B68D	211591	27714	6598	770	3409	5939	330
		CE71	78921	5902	7589	0	2024	1180	0

Rectal autopsy	Unadjuvanted	A730	46800	111413	16908	5737	1208	24758	906
		9B7A	6711	2237	31320	0	15660	0	0
		55A3	529880	1328	5312	19920	172643	27888	18592
		A50D	24167	166667	85000	39167	21667	22500	8333
		D2CD	49645	239559	8668	6304	1576	15760	788
		94D5	441860	17442	0	23256	145349	63953	23256
	IL-4R antagonist	DD1B	0	0	63830	10638	63830	0	0
		ECEB	608579	0	2681	8043	80429	8043	5362
		AAB5	481879	1342	5369	5369	120805	6711	5369
		9CBC	5057	8428	8428	9692	1264	843	1264
		DA45	*						
		F068	43731	58792	54981	13246	10343	9073	3448
	Empty vector	B68D	8323	39834	9512	44590	4756	17836	4162
		CE71	*						

[#]Please note that the table layout corresponds to the figure layout.

Supplementary Table 4: Absolute Gag-specific rectal $CD8^+$ T cell counts (per 10^6) corresponding to Figure 7b[#]

		Animal ID	IL-2+	IFN-γ+	TNF+	TNF+ IFN-γ+	IL-2+ TNF+	IFN-γ+ IL-2+	IFN-γ+ TNF+ IL-2+
Rectal biopsy	Unadjuvanted	A730	5694	2569	3749	0	208	555	0
		9B7A	2574	1287	3717	0	0	0	0
		55A3	4429	1661	3876	185	185	185	0
		A50D	4271	6406	3537	1001	67	67	0
		D2CD	10470	3125	7032	156	156	469	0
		94D5	5348	2741	3371	0	90	90	0
	IL-4R antagonist	DD1B	4303	5635	2869	102	0	0	0
		ECEB	9323	4351	3729	0	622	2486	0
		AAB5	2255	2481	2650	0	169	56	0
		9CBC	7045	13097	4634	284	331	331	0
		DA45	10793	7759	5925	635	353	141	0
		F068	2097	2719	1554	0	155	0	0
	Empty vector	B68D	5173	4456	3329	102	205	359	0
		CE71	4276	5248	3110	428	194	117	0
Rectal autopsy	Unadjuvanted	A730	50596	5963	4782	1712	1476	3365	1004
		9B7A	362	12114	723	1266	723	542	542
		55A3	45155	941	25400	0	18815	941	0
		A50D	2329	10647	54567	2204	707	790	125
		D2CD	191617	0	23952	11976	17964	89820	11976
		94D5	15513	985	7880	985	2216	2709	739
	IL-4R antagonist	DD1B	3906	3906	3906	0	3906	0	0
		ECEB	30371	408	14880	1427	11822	2038	1019
		AAB5	17773	671	46781	1844	9557	2347	1341
		9CBC	2875	15451	30902	719	1437	0	0
		DA45	*						
		F068	3104	8669	18624	833	265	454	151
	Empty vector	B68D	2964	9634	48172	1729	2964	3211	1482
		CE71	*						

[#]Please note that the table layout corresponds to the figure layout.

Supplementary Table 5: Absolute Gag-specific cervico-vaginal CD4⁺ (top) and CD8⁺ (bottom) T cell counts (per 10^6) corresponding to Figure 8#

		Animal ID	IL-2+	IFN-γ+	TNF+	TNF+ IFN-γ+	IL-2+ TNF+	IFN-γ+ IL-2+	IFN-γ+ TNF+ IL-2+
CD4⁺ T cells	Unadjuvanted	A730	72976	9122	0	2281	0	12543	0
		9B7A	♂						
		55A3	♂						
		A50D	71734	33191	3212	1071	1071	18201	1071
		D2CD	♂						
		94D5	231579	10526	10526	31579	63158	63158	21053
	IL-4R antagonist	DD1B	*						
		ECEB	435811	3378	6757	37162	72635	47297	25338
		AAB5	291045	2488	2488	29851	59701	37313	27363
		9CBC	15989	76423	25745	7859	2710	21409	1897
		DA45	♂						
		F068	♂						
	Empty vector	B68D	10055	25138	17597	2011	1508	5028	0
		CE71	♂						

CD8⁺ T cells	Unadjuvanted	A730	4362	3216	1528	860	509	509	287
		9B7A	♂						
		55A3	♂						
		A50D	16183	4737	4144	3355	2171	1974	789
		D2CD	♂						
		94D5	6567	938	31895	938	0	0	0
	IL-4R antagonist	DD1B	4054	0	0	0	8108	0	0
		ECEB	278290	188	1883	1695	11486	1883	1506
		AAB5	52055	783	1174	783	1957	1566	391
		9CBC	637	42775	1273	509	0	127	0
		DA45	♂						
		F068	♂						
	Empty vector	B68D	0	8665	1617	0	0	0	0
		CE71	♂						

#Please note that the table layout corresponds to the figure layout.
